# A *SmelAAT* Acyltransferase Variant Causes a Major Difference in Eggplant (*Solanum melongena* L.) Peel Anthocyanin Composition

**DOI:** 10.3390/ijms22179174

**Published:** 2021-08-25

**Authors:** Francesco Elia Florio, Stefano Gattolin, Laura Toppino, Laura Bassolino, Marta Fibiani, Roberto Lo Scalzo, Giuseppe Leonardo Rotino

**Affiliations:** 1 CREA, Research Centre for Genomics and Bioinformatics, 26836 Montanaso Lombardo, Italy; francesco.florio@unimi.it (F.E.F.); laura.toppino@crea.gov.it (L.T.); laura.bassolino@crea.gov.it (L.B.); 2 Department of Agricultural and Environmental Sciences (DISAA), University of Milan, 20133 Milan, Italy; 3 CNR—National Research Council of Italy, Institute of Agricultural Biology and Biotechnology (IBBA), 20133 Milan, Italy; 4 CREA, Research Centre for Cereal and Industrial Crops, 40128 Bologna, Italy; 5 CREA, Research Centre for Engineering and Agro-Food Processing, 20133 Milan, Italy; marta.fibiani@crea.gov.it (M.F.); roberto.loscalzo@crea.gov.it (R.L.S.)

**Keywords:** *Solanum melongena*, eggplant, anthocyanin, fruit coloration, marker-assisted selection (MAS), delphinidin-3-rutinoside, nasunin

## Abstract

Eggplant berries are rich in anthocyanins like delphinidin-3-rutinoside (D3R) and nasunin (NAS), which are accumulated at high amounts in the peel. NAS is derived by D3R through acylation and glycosylation steps. The presence of D3R or NAS is usually associated with black-purple or lilac fruit coloration of the most cultivated varieties, respectively. Building on QTL mapping position, a candidate gene approach was used to investigate the involvement of a BAHD anthocyanin acyltransferase (*SmelAAT*) in determining anthocyanin type. The cDNA sequence comparison revealed the presence of a single-base deletion in D3R-type line ‘305E40’ (*305E40_aat*) with respect to the NAS-type reference line ‘67/3’. This is predicted to cause a frame shift mutation, leading to a loss of *SmelAAT* function and, thus, D3R retention. RT-qPCR analyses confirmed *SmelAAT* and *305E40_aat* expression during berry maturation. In D3R-type lines, ‘305E40’ and ‘DR2’, overexpressing the functional *SmelAAT* allele from ‘67/3’, the transcript levels of the transgene correlated with the accumulation of NAS in fruit peel. Furthermore, it was also found a higher expression of the transcript for glucosyltransferase *Smel5GT1*, putatively involved with *SmelAAT* in the last steps of anthocyanin decoration. Finally, an indel marker matching with anthocyanin type in the ‘305E40’ × ’67/3’ segregating population was developed and validated in a wide number of accessions, proving its usefulness for breeding purposes.

## 1. Introduction

Anthocyanins are a class of flavonoids playing important roles in promoting plant pollination and seed dispersal and protecting against biotic and abiotic stress [1]. They are also responsible for the blue, purple, and red coloration of many plant tissues. Besides their importance for the plant, anthocyanins have long been considered valuable secondary metabolites for their potential as natural food coloring agents and their beneficial effect on human health as antioxidants [2,3,4,5]. Anthocyanins are synthetized in plants through a branching of the phenylpropanoid pathway, which uses phenylalanine as precursor; this network is highly conserved among species and one of the most studied in eudicots [6,7]. In dicotyledonous species, the structural genes encoding enzymes involved in the biosynthetic pathway are commonly divided into two clusters: the Early Biosynthetic Genes (EBGs) and the Late Biosynthetic Genes (LBGs) [8]. In the first part of the pathway, through the action of the EBGs, the metabolic precursors phenylalanine and *p*-coumaroyl-CoA are processed to produce naringenin [9]. Naringenin is then converted to dihydrokaempferol, a dihydroflavonol, the last compounds synthesized through the action of the EBGs [10]. The enzymatic steps catalyzed by LBGs begin with the action of DIHYDROFLAVONOL 4-REDUCTASE (DFR), which converts dihydroflavonols to leucoanthocyanidins. Then, ANTHOCYANIN SYNTHASE (ANS) originates anthocyanidins such as pelargonidin, cyanidin, peonidin, delphinidin, petunidin, and malvidin [11]. Anthocyanidins are converted into anthocyanins by the species-specific addition of glycosyl moieties, often initiated by an O-glycosylation at the 3 position and by further modifications, such as aromatic acylation [12].

*Solanum melongena* L., usually known as common eggplant, belongs to the *Solanaceae* family and is an important vegetable crop cultivated worldwide. The eggplant berry has been recognized as an important source of phenolic compounds, mainly represented by chlorogenic acid and anthocyanins in the flesh and peel, respectively [13]. Due to a growing interest in the nutraceutical properties of vegetables rich in secondary metabolites, the accumulation profile of these compounds has been included as a target of eggplant breeding programs and of studies on the genetic regulation of anthocyanin biosynthesis [6,13,14,15,16,17,18]. Typically, eggplant fruits produce the anthocyanidin delphinidin, which, in different accessions, is converted into the conjugated anthocyanins delphinidin-3-rutinoside (D3R) or delphinidin-3-(*p*-coumaroylrutinoside)-5-glucoside, known as nasunin (NAS). In other species, these last steps of decoration are controlled by the action of an ACYLTRANSFERASE (AAT) that uses *p*-coumaroyl-CoA as a donor to acylate the rutinose residue and of a GLUCOSYLTRANSFERASE (5GT) adding a glucose moiety in the 5 positions [19,20,21,22,23]. As a result of the presence of these two anthocyanin types, eggplant peel color varies from black/dark purple to dark/light lilac, generally reflecting the presence of D3R or NAS, respectively [19,24]. NAS is typically found in ‘Type 1’ (Japanese type) eggplants while the ‘Type 2’ (non-Japanese type) accessions are characterized by the D3R presence [23]. ‘Type 2’ cultivars with intense, blackish-purple colored fruits are widespread worldwide and are particularly important for the Mediterranean and North American markets [25]. The anthocyanin profile in eggplant was long thought to be a complex trait, involving several loci with assumed epistatic interactions and/or pleiotropic effects [19,26]. QTL-mapping studies using segregating progenies from the cross between ‘305E40’ and ’67/3’ lines [24,27,28] and GWAS approaches [29,30] allowed the identification of two QTLs’ clusters spanning genomic regions on the E05 and E10 chromosomes involved in anthocyanin pigmentation of flowers, vegetative organs, and fruit peel. QTLs on E10 appear mainly involved in anthocyanin intensity and distribution in all vegetative tissues as well as in peel color under the fruit calyx. Conversely, QTLs on E05 are involved in the pigmentation of corolla and fruits, as well as in the alternative production of D3R or NAS in the fruit peel. 

**Figure 1:**
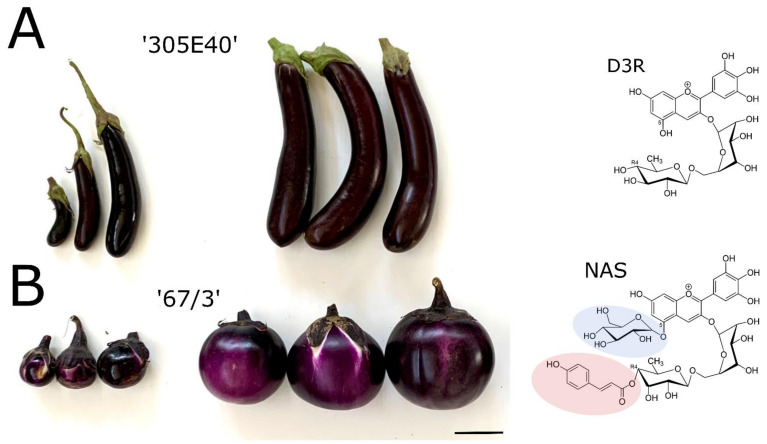
Phenotypic features of ‘67/3’ and ‘305E40’ fruits. (**A**) Berries of ‘305E40’ (purple) and chemical structure of delphinidin-3-rutinoside (D3R). (**B**) Berries of ‘67/3’ (lilac) and chemical structure of delphinidin-3-[*p*-coumaroylrutinoside]-5-glucoside (NAS). The differences between D3R and NAS are shaded in pink (*p*-coumaroyl acylation of the rutinose residue) and in blue (5-O-glucosylation). For each eggplant line: on the left, young unripe berries (stage A); on the right, commercially mature berries (stage B); scale bar: 5 cm.

More recently, a RIL population from the intraspecific cross between ‘305E40’ × ‘67/3’ was employed for developing a GBS-based, high-density linkage map and locating QTLs for many fruits traits, including shape and anthocyanin tonality [31]. The same population was recently characterized for the metabolic composition of the fruit peel and flesh, allowing the discovery and location of mQTLs for metabolic compounds belonging to flavonoid, glycoalkaloid, and polyamine classes [32]. In particular, a QTL cluster on E05 was depicted to control, in an opposite way, the levels of D3R and NAS, the two alternative anthocyanin forms characterizing the parental lines of the RIL population. Genome annotation in combination with sequence differences in the two parental lines supplied a key tool to gather valuable information for QTL fine mapping and for identification of some valuable candidates controlling the synthesis of the considered metabolites. Among them, the gene *Smel_005g236240* encoding for a BAHD anthocyanin acyltransferase (hereafter referred to as *SmelAAT*) was revealed as the most promising candidate for the conversion of D3R into NAS, as the SnpEff analysis performed on the two parental lines revealed a variant that could determine a severe effect in the CDS sequence of the ‘305E40’ parent, in agreement with the biochemical data [32]. In the present work, we used a candidate gene approach to investigate the role of *SmelAAT* in the differential accumulation of D3R or NAS in lines ‘305E40’ and ‘67/3’, respectively (Figure 1). A loss-of-function indel variant detected in ‘305E40’ (hereafter, *305E40_aat*) with respect to the *SmelAAT* coding sequence is likely causative of the retention of D3R in this line. NAS accumulation was evaluated in two D3R-producing lines (‘305E40’ and ‘DR2’) when transformed to express the *SmelAAT* coding sequence from ‘67/3’, thus assessing the pivotal role of this gene in eggplant anthocyanin decoration. 

A high-resolution melting (HRM) indel marker was developed on the candidate *SmelAAT* variants and employed for validation in a wide number of eggplant accessions.

## 2. Results

### 2.1. Identification of a Candidate Gene for NAS or D3R Accumulation

**Figure 2:**
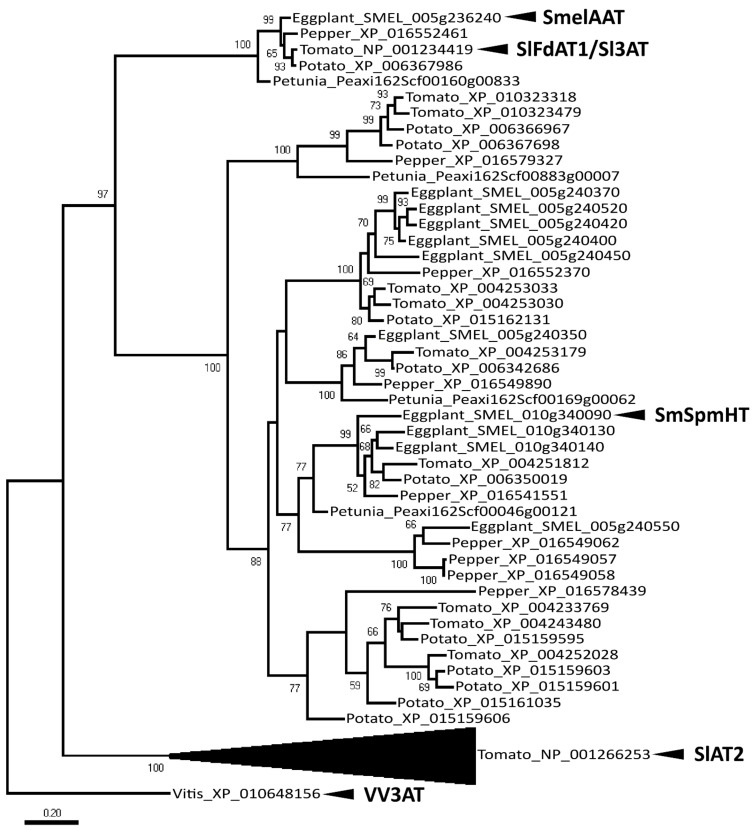
Evolutionary relationships among acyltransferase proteins in different plant species. The tree was obtained using peptide sequences from *S. melongena* (Eggplant), *S. lycopersicum* (Tomato), *S. tuberosum* (Potato), *C. annuum* (Pepper), *P. axillaris* (Petunia), and *Vitis vinifera* (Vitis). SmelAAT and other acyltransferases with known function from the literature (SlAT2, SmSpmHT, SlFdAT1/Sl3AT) are indicated next to the corresponding sequences. A previously described *V. vinifera* sequence (corresponding to Vv3AT) was included as the outgroup. A subtree comprising the 32 most distantly related sequences to SmelAAT was compressed for clarity. Only bootstrap values >50 are shown.

Among gene models annotated within the QTL confidence interval mapped on chromosome E05, SMEL_005g236240, encoding a putative Acetyl-CoA-benzylalcohol acetyltransferase (SmelAAT), was a prime candidate for anthocyanin tonality and NAS/D3R alternative accumulation. To elucidate the function of SmelAAT among eggplant acyltransferases, the phylogenetic relationship of protein sequences from S. *melongena* and other Solanaceae was analyzed. A blast search of the SmelAAT sequence (from ‘67/3’, GeneBank submission Id 2424224) against the ‘67/3’ eggplant genome in NCBI and Sol Genomics databases allowed us to identify putative acyltransferase amino acid sequences from *S. melongena*, *Solanum lycopersicum*, *Solanum tuberosum*, *Capsicum annuum*, and *Petunia axillaris*, which were used to obtain a molecular phylogenetic tree (Figure 2, File S1).

Some of the sequences analyzed were identified as corresponding to previously described BAHD acyltransferases [33] with known function: SlAT2 (NP_001266253, Solyc01g105580), capable of acyl sucrose acetylation [34], SmSpmHT (SMEL_010g340090) with spermine hydroxycinnamoyl transferase activity [35], and SlFdAT1 (NP_00134419, Solyc12g088170), acting as a flavonoid-3–O-rutinoside-4′′′–O-phenylacyl transferase [36].

The sequence of SlFdAT1 corresponds to that of Sl3AT [37], which was used in in vivo experiments confirming that this enzyme can use cyanidin 3-O-rutinoside as substrate to produce cyanidin 3-O-(4′′′-O-(*p*-coumaroyl) rutinoside) in transgenic tobacco. Phylogenetic analysis placed SmelAAT in a small clade comprising a single sequence derived from each of the considered species; among them, SlFdAT1 showed the strongest in vitro activity using *p*-coumaroyl-CoA as donor and D3R as acceptors [36]. The existence of this small clade separated from other anthocyanin phenylacyl transferases within the Solanaceae, and the relatedness of SmelAAT with SlFdAT1 suggests a conserved biochemical function, supporting its potential role in D3R to NAS conversion.

### 2.2. Identification of a Homozygous Loss-of-Function SmelAAT Allele in ‘305E40’

**Figure 3:**
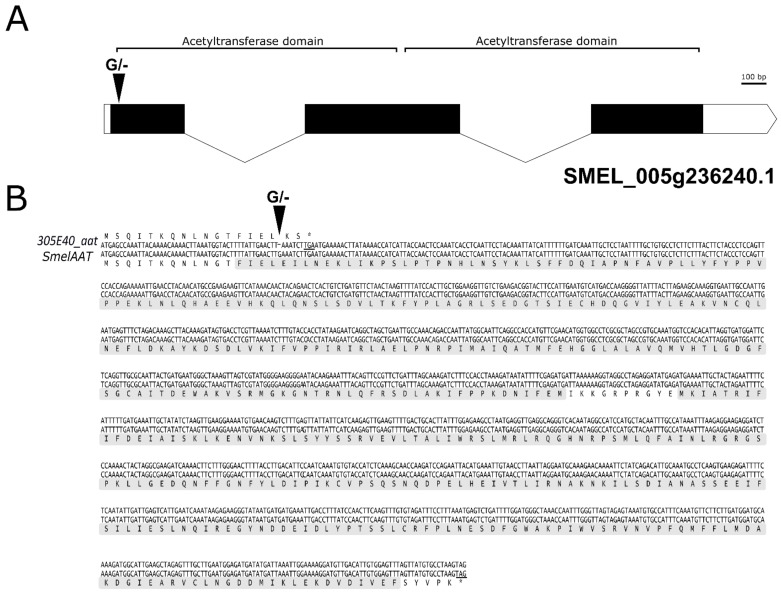
Sequence of the *S. melongena 305E40_aat* variant and SmelAAT genes. (**A**) Gene structure of SMEL_005g236240.1 (*SmelAAT*). Black solid boxes indicate CDS exons, white boxes indicate UTR regions. Gene portions encoding for the acetyltransferase domains are shown. The arrowhead indicates the position of the identified single-base G deletion. (**B**) Comparison of the CDS and translated amino acidic sequences of *SmelAAT* and the *305E40_aat* allele. Acetyltransferase domains are shadowed.

The presence of polymorphisms within the AAT coding sequences was investigated by comparing full length cDNA from peel samples of ‘67/3’ and ‘305E40’, a NAS (Type-1) and a D3R (Type-2) line, respectively. Sequence comparison revealed a single polymorphism consisting of a homozygous nucleotide deletion in the ‘305E40’ *SmelAAT* allele (hereafter 305E40_aat) at position 49/1359 of the coding sequence, resulting in a frameshift and premature stop codon in the predicted protein (Figure 3, File S2). Premature termination of the predicted peptide occurs after 18 amino acids, causing the lack of both CoA-dependent acyltransferase domains (CATH Domain 2bghA01). Therefore, this mutation would give rise to a null allele abolishing the SmelAAT function and NAS biosynthesis in ‘305E40’.

### 2.3. Complementation of SmelAAT Induces NAS Production in “Type 2” Genotypes Homozygous for the 305E40_aat305E40_aat Allele

To confirm the involvement of SmelAAT in NAS biosynthesis and support the hypothesis of a loss-of-function mutation in the 305E40_aat allele as the cause for the lack of D3R into NAS conversion in ‘305E40’ line, a transgenic complementation was performed. Two D3R-producing lines homozygous for the *305E40_aat* allele (‘305E40’ and ‘DR2’), producing exclusively D3R with no presence of NAS, were complemented by overexpressing the coding sequence of *SmelAAT* from the ‘67/3’ line under the constitutive promoter 35S. HPLC analysis of fruit anthocyanin profiles showed significantly different amounts of NAS accumulated among the 12 independent T1 overexpressing lines (Table 1), demonstrating that expression of the *SmelAAT* functional allele partially restores the D3R to NAS conversion in both ‘DR2’ and ‘305E40’ backgrounds. Three ‘DR2’ overexpressing plants accumulated, in the analyzed fruits, very low amounts of NAS, which were statistically not different from that of ‘DR2’. In the fruit peels of the remaining eight ‘DR2’ plants, a noticeable accumulation of NAS was detected, similar to that previously found in ‘67/3’, the line donor of the *SmelAAT* sequence (Table 1). With regard to D3R accumulation, all the ‘DR2’ overexpressing lines accumulated significantly less D3R than the control, except the DR2 #3-1 plant, and simultaneous presence of both NAS and D3R with variable contents was evidenced in most of the overexpressing lines.

**Figure 4:**
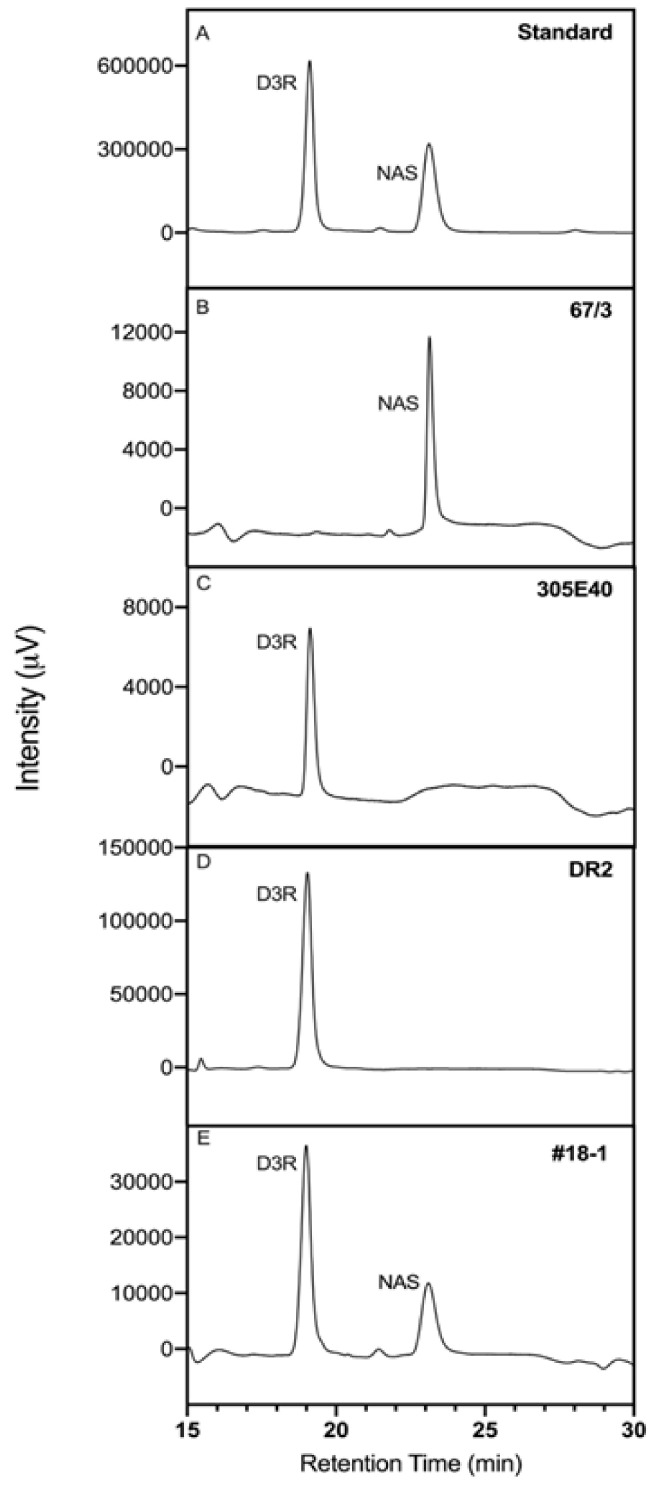
HPLC chromatograms of anthocyanin types. (**A**) D3R and NAS standards and four peel sample extracts from lines (**B**) ‘67/3’, (**C**) ‘305E40’’, (**D**) ‘DR2’, and (**E**) ‘DR2’-*p35S::SmelAAT* #18-1 recorded at 525 nm.

**Figure 5:**
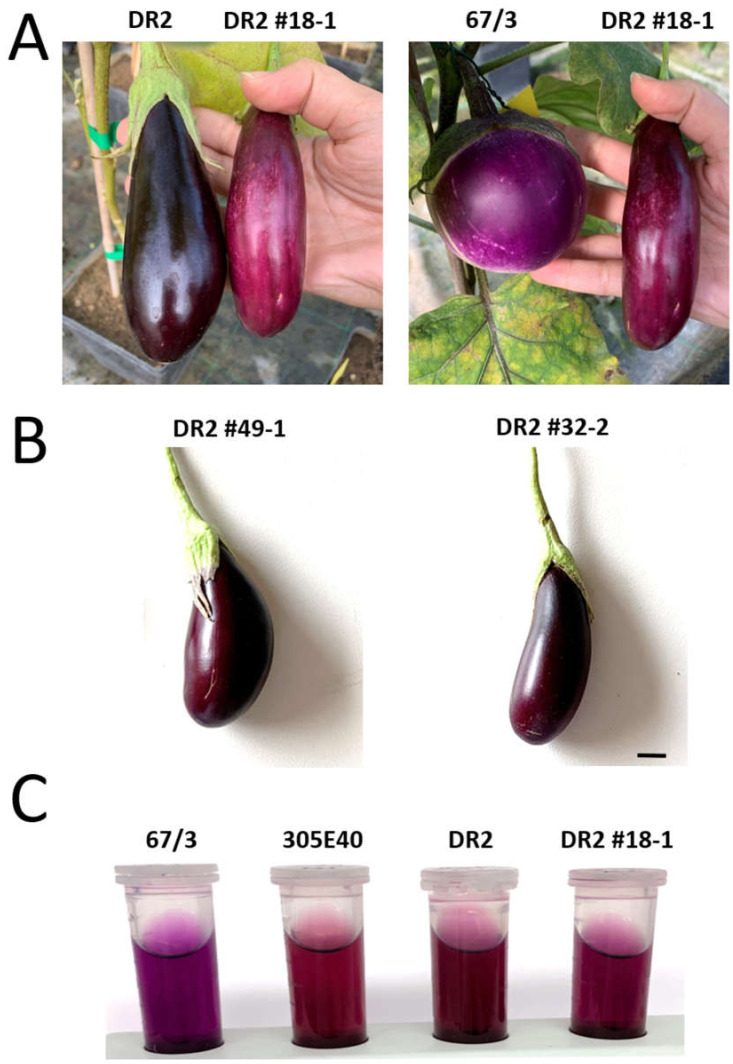
Peel color of *p35S:SmelAAT* fruits. (**A**) Wild-type DR2 and ‘67/3’ fruits compared with a fruit of line ‘DR2’ #18-1. (**B**) Fruits of two other transformed lines ‘DR2’ #49-1 and ‘DR2’ #32-2. (**C**) Comparison of ethanol anthocyanin extracts obtained from fruit peel of ‘67/3’, ‘305E40’, DR2, and line ‘DR2’ #18-1. In (**B**), scale bar = 1 cm.

Moreover, consistent with its highest percentage (45%) of NAS over total anthocyanins among lines (Table 1, Figure 4), the peel color of DR2 #18-1 fruits also resulted as noticeably different from that of ‘DR2’, acquiring tones intermediate between the pigmentation of ‘DR2’ and ‘67/3’ (Figure 5A), while in other lines the difference in color resulted as more subtle (Figure 5B). This difference was also observable in ethanol anthocyanin peel extracts (Figure 5C).

Five ‘DR2’ *p35S::SmelAAT* T1 lines, selected according to their different NAS content (see Table 1), were used to analyze allele-specific expression of native *305E40_aat* and transgenic *p35S::SmelAAT* in peel samples.

**Figure 6:**
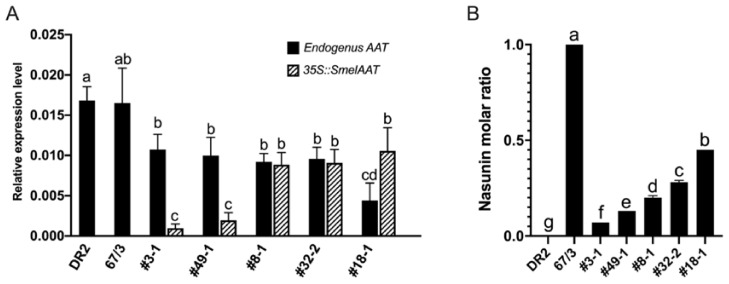
Overexpression of *p35S::SmelAAT* in 305E40_aat ‘DR2’ genotype. (**A**) RT-qPCR expression analysis of *SmelAAT* (in line ‘67/3’), *305E40_aat* and *p35S::SmelAAT* in eggplants’ peel samples of ‘DR2’ and five T1 transformants of ‘DR2’. Data are the means of three biological replicates ± SD. (**B**) NAS molar ratio over the total amount of anthocyanin (NAS+D3R) in peel of ‘DR2’, ‘67/3’, and the five *p35S::SmelAAT* transformants. Different letters in (**A**) and (**B**) indicates significant differences at *p* < 0.05 (Tukey’s HSD).

Interestingly, the expression levels of native *305E40_aat* and *SmelAAT* transcripts in ‘DR2’ and ‘67/3’ were not significantly different (Figure 6A), suggesting that, despite the missense-causing mutation, the *305E40_aat* transcript does not undergo nonsense mediated decay [38]. The expression levels of *p35S::SmelAAT* exhibited a certain variability among the five transgenic lines showing lower expression levels in #3-1 and #49-1 plants and the highest in #18-1. Conversely, transcript abundance of the native *305E40_aat* gene in the transgenic lines negatively correlated with the *p35S::SmelAAT* levels (R = −0.59). By comparing the percentage of NAS on the total anthocyanin molar amount (i.e., the sum of D3R and NAS) in ‘DR2’, ‘67/3’, and the five T1 lines (Figure 6B), an almost linear correlation between the increasing levels of NAS and the expression levels of *p35S::SmelAAT* was found (R = 0.91). Taken together, these results demonstrated a pivotal function of SmelAAT in NAS biosynthesis and a plausible causative role of the *305E40_aat* allele in D3R retention in ‘305E40’ and ‘DR2’.

### 2.4. The Expression of Functional SmelAAT Increases the Transcription of Smel5GT1

The conversion of D3R to NAS requires two subsequent steps of anthocyanin decoration [19]. In petunia, a 5GT (PH1, GenBank: BAA89009.1) is responsible for the 5-O-glucosylation taking place after the acylation of the rutinose residue with the *p*-coumaroyl moiety [39]. A homology search in the eggplant genome using the petunia 5GT peptide sequence as bait allowed us to identify SMEL_005g238370 as the gene encoding for the eggplant *Smel5GT1* orthologue (88% identity at the AA level). Expression levels of *SmelAAT* and *Smel5GT1* in different tissues were evaluated in previously published ‘67/3’ RNA-seq data [40], which included fruit tissues at different ripening stages but not fruit peel alone. The relative expression levels suggested a similar pattern for *SmelAAT* and *Smel5GT1* in the eggplant berries. As eggplant fruits produce anthocyanins in the peel at both ripening stages, A (young unripe) and B (commercial mature) (Figure 1), the expression levels of AAT (intended as *SmelAAT* and/or *305E40_aat*) and *Smel5GT1* in the fruit peel of these stages were investigated by comparing them in the genetic backgrounds ‘67/3’, ‘305E40’, and ‘DR2’, and the transformed T1 plants ‘DR2’ #18-1, #32-2, and #49-1. 

**Figure 7:**
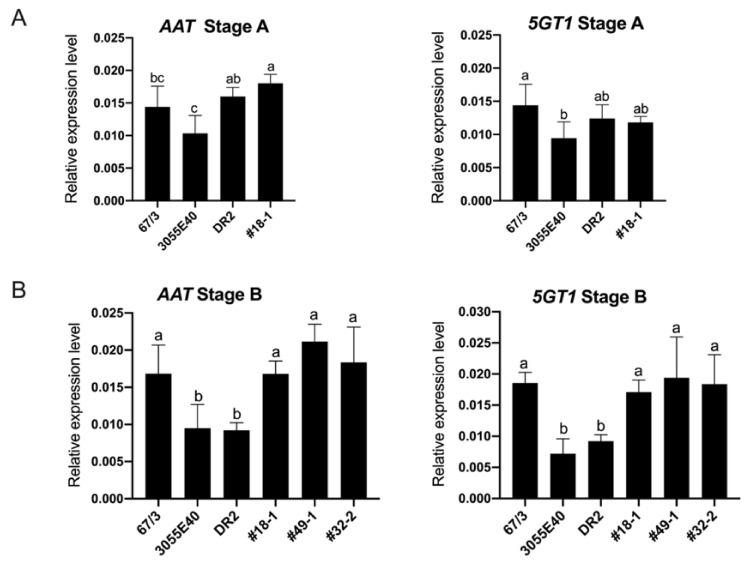
RT-qPCR transcription profiling of *SmelAAT* and *Smel5GT1* in peels at stages A and B of fruit ripening. Expression levels in lines ‘67/3’, ‘305E40’, and ‘DR2’ were compared to those of ‘DR2’ *p35S::SmelAAT* #18-1 at stage A (**A**) and of transgenic plants ‘DR2’ *p35S::SmelAAT* #18-1, #49-1, and #32-2 at stage B (**B**). Data are the means of three biological replicates ± SD. Different letters indicate significant differences at *p* < 0.05 (Tukey’s HSD).

At ripening stage A (Figure 7A), the *AAT* transcripts’ levels were comparable between ‘DR2’ and the transformed T1 plant and similar to that of *SmelAAT* in ‘67/3’, while the *305E40_aat* transcript resulted to be lower in ‘305E40’ than in ‘DR2’.

Expression of *Smel5GT1* at this stage was lower in ‘305E40’ than in ‘67/3’ and ‘DR2’, while in the plant ‘DR2’ #18-1 it was similar to the other three untransformed genotypes. At stage B, the expression of *SmelAAT* in ‘67/3’ resulted as increased compared to *305E40_aat* in ‘305E40’ and ‘DR2’ but was comparable to the AAT transcript levels in ‘DR2’ #18-1, #49-1, and #32-2 (Figure 7B). Interestingly, the same expression profile was observed for the *Smel5GT1*, suggesting that the presence of p35S-driven *SmelAAT* transcript translates into a concurrent increase in the expression of Smel5GT1 in the transformed plants, compared to the ‘DR2’ and ‘305E40’ lines. 

### 2.5. An AAT-HRM Indel Marker Correlates with the NAS/D3R Phenotype

In light of the evidence around the two AAT allelic variants as causative of the differential accumulation of anthocyanin types, a genotyping tool was developed for molecular-assisted breeding purpose. A HRM marker (AAT-HRM) was designed around the G nucleotide indel variant within the coding sequence of the gene (Primers in Table S1). Melt analysis revealed the presence of distinguishable single peaks differentiating the *SmelAAT allele* of *SMEL_005g236240* present in the sequenced genome of ‘67/3’ from the *305E40_aat* variant discovered in ‘305E40′, with heteroduplex formation in the heterozygous F1 hybrid (Figure S1). The ability of this marker to predict the presence of NAS or D3R was firstly tested in 84 F2 individuals from the cross ‘305E40’ × ‘67/3’ (Table S2), for which visual phenotypical evaluation of peel color and biochemical data analysis of NAS/D3R anthocyanin composition were already available [24]. The AAT-HRM marker showed a perfect correlation with the F2 biochemical phenotypes and a 3:1 segregation ratio (chi-square = 5.2143), as confirmed by the allelic dominance of *SmelAAT* over *305E40_aat,* associated with the presence of NAS in heterozygous F1 hybrids [24]. This analysis also allowed us to test the effectiveness of the visual scoring of peel color phenotype commonly used in eggplant selection, revealing that, with respect to both molecular and biochemical scoring, only 4/84 F2 individuals were phenotypically mis-annotated in this population, due to fruits characterized by a particularly dark skin color and, therefore, difficult to distinguish between blackish lilac and blackish purple. We then tested the AAT-HRM marker on further 61 independent accessions belonging to the CREA-GB germplasm collection, for which phenotypic visual evaluation of peel color was available [30] (Table 2). 

For 10 of these, anthocyanin compositions had also been recorded previously [13]. We found a high correlation (R = 0.87) between the HRM haplotype and the visual peel color phenotype. Running an additional HPLC analysis on the accession, for which the blackish peel color made it particularly difficult to score them as lilac or purple, enabled us to correct their phenotypic data according to the biochemical results, and the correlation between marker and phenotype further increased to R = 0.91. These results showed that the *305E40_aat* allele is widespread in the tested germplasm and confirmed the suitability of the AAT-HRM marker for the selection of genotypes with different anthocyanin pigmentation in breeding programs.

## 3. Discussion

Characterization of the diversity in anthocyanin forms available from various plant sources has gained increasing interests in the last years, in light of their potential health-promoting activity and their use as alternatives to synthetic food colorants (for reviews see [5,41,42]). The peel of *S. melongena* berries accumulates high amounts of anthocyanins, and their chemical nature is reflected in the color they confer, differentiating the two most common typologies of cultivated eggplant. To date, the genetic determinism behind the distinct forms of accumulated anthocyanins, D3R or NAS, is still unknown.

In this study, a mutation in the coding sequence of *SmelAAT* was identified as a putative variant responsible for the difference in anthocyanin type between ‘67/3’ and ‘305E40’ (Figure 1), two lines belonging, respectively, to “Type 1” (NAS) with a lilac peel color and “Type 2” (D3R) with a purple coloration. Phylogenetic analysis of sequences identified by homology and available functional data suggests that *SmelAAT* encodes a BAHD acyltransferase able to acylate anthocyanins using acyl-CoA donors. The close proximity of SmelAAT to SlFdAT1/Sl3AT [36,37] within a small subclade that evolved in *Solanaceae* (Figure 2) indicates that SmelAAT may acylate D3R using *p*-coumaroyl-CoA as a donor in the pathway leading to NAS biosynthesis as the final anthocyanin compound. The identification of a 1bp G deletion within the *SmelAAT* coding sequence, putatively causing a loss-of-function mutation (i.e., a frame shift and premature termination of the predicted protein) in the D3R lines ‘305E40’ and ’DR2’, suggested a pivotal role of *SmelAAT* in the biosynthesis of NAS from D3R. Indeed, overexpression of the ‘67/3’ *SmelAAT* allele in both ‘305E40’ and ‘DR2’ backgrounds, homozygous for the *305E40_aat* allele, resulted in a well detectable conversion of D3R to NAS in most lines (Table 1), confirming that expression of the putatively functional *SmelAAT* is sufficient to restore acylation and glycosylation activities. Most of the transgenic ‘DR2’ plants accumulated significantly less D3R than their control and, interestingly, revealed the presence of both anthocyanin types with levels of NAS comparable to that detected in the NAS-type ‘67/3’ (Table 1). To the best of our knowledge, the simultaneous presence of both D3R and NAS in eggplant peel has never been reported before or represents a very rare event in eggplant peels [13,23], and this influenced the appearance of the berries of the transgenic plants, which looked different from those of both ‘DR2’ and ‘67/3’ (Figure 5). Interestingly, three *p35S::SmelAAT* plants (i.e., DR2 #14-1, #51-1, and #6-3) displayed very weak accumulation of both NAS and D3R. A possible explanation for this could be transgene co-suppression due to multiple insertions and/or that the site of insertion of the transgene(s) in the genome [43] could somehow negatively affect the pathway responsible for the accumulation of the two anthocyanins. These materials may be exploited in further studies to better understand the genetic regulation of anthocyanin accumulation in eggplant peel. As NAS formation requires a 5-O-glucosilation step in addition to the *p*-coumaroyl acylation of the rutinoside (Figure 1), we decided to investigate whether the expression of the 5-O-glucosyltransferase putatively responsible for the completion of NAS production was affected in transgenic plants. The *S. melongena* orthologue (*Smel5GT1*, *SMEL_005g238370*) of the gene responsible for this step in petunia [39] was, therefore, identified by a homology search in the ‘67/3’ genome. We evidenced that the partial restoration of *SmelAAT* function in the transgenic lines was accompanied by an increase in the transcript of *Smel5GT1* to levels comparable to those in ‘67/3’ (Figure 7), suggesting an influence of substrate on the transcription of this gene. These results are consistent with a scenario in which the presence of the exogenous *SmelAAT* allele promotes DR3 acylation in ‘DR2’, providing a suitable substrate for *Smel5GT1*, which, in turn, triggers an increase in its expression. This would be in accordance with Yamazaki et al. [39], reporting that in petunia the acylation of the rhamnose group is a required step prior to 5-O-glucosylation. Taken together, RT-qPCR data and the absence of peaks ascribable to glucosylated forms of D3R in the HPLC profiles of ‘305E40’ and ‘DR2’ fruit peel provide evidence that the same is plausibly true also for eggplant. The 5GT function seems to have evolved to increase the solubility of the acylated anthocyanins. Flavonoid 3-O-(4’’’-O-(*p*-coumaroyl)-rutinoside) has been shown to precipitate at slightly low pH to form Anthocyanic Vacuolar Inclusions (AVI), and 5-O-glycosylation increases solubility and reduces any negative effects that discrete solid structures in the vesicles and the vacuole could cause [37]. While *SmelAAT* transgenic complementation unequivocally demonstrates the functional role of this gene in NAS biosynthesis, it also provides strong evidence that the indel mutation is causative of the phenotypic variation in anthocyanin accumulation. Interestingly, this mutation did not appear to compromise the stability of the *305E40_aat* transcript in ’305E40’ and ’DR2’ lines. While cDNA sequencing ruled out alternative splicing events, it is possible that an alternative start site downstream of the mutation could still lead to translation of the transcript, preventing nonsense-mediated decay [38]. In silico analysis suggests that this could potentially give rise to a 299-aa-long peptide, starting from methionine 154 of *SmelAAT* (Figure 3). This protein, however, would lack 141 of the 210-aa-long first CATH domain, suggesting a hindered acyltransferase activity, as supported by the absence of NAS accumulation in homozygous genotypes for this allele.

Genotyping of 84 individuals of a ‘67/3′ × ‘305E40′ F2 population with an AAT-HRM marker (Table S1) confirmed perfect correspondence of the different allelic forms (AA, aa, and Aa) with the anthocyanin identified by HPLC analysis (NAS or D3R). Furthermore, the genic marker proved to be strongly associated (R = 0.91) with the phenotype of additional 61 accessions (Table 2). This makes it a valuable predictive molecular tool for marker-assisted breeding programs aimed at maintaining an anthocyanin type in the progeny. This tool may be exploited to foresee eggplant fruit coloration, which is among the key determinants influencing consumer choices. As seen above, discrepancies between the visually scored anthocyanin tonality, commonly used to phenotype and/or to select for peel coloration, and anthocyanin type are due to difficulties in differentiating lilac NAS-containing from purple D3R-containing fruits in lines or accessions with a particularly dark peel color. In fact, the color perceived by the human eye is influenced by the combined effect of several concurrent factors, which include but are not limited to the anthocyanin type and content. The optical properties of the plant cell and tissue structures, vacuole pH, co-pigmentation, formation of super complexes, and AVIs, association with metals, are features determining the huge diversity of colors in plants [44] and most of them are likely operating in eggplant as well. Moreover, the presence, in some eggplant accessions, of a layer of chlorophyll-containing tissue located between the peel and the outermost part of the flesh [28] could also account for further difficulties in distinguishing between blackish-lilac (NAS) and blackish-purple (D3R) fruits. This could also be the case for our overexpressing lines, in which the visual determination of the presence of NAS was hindered not only by the presence of the green layer under the peel but also by the contemporaneous presence of both D3R and NAS. HPLC analysis unambiguously allowed us to determine the anthocyanin type also increasing the reliability of the HRM marker to discriminate between ‘Type1’ and ‘Type2’ accessions. The finding that the difference in color between ‘Type1-NAS’ and ‘Type2-D3R’ accessions is due to or strongly influenced by the onset of a loss-of-function allele and the identified variant is the same in the majority of the tested germplasm accessions (Table 2) suggests that the *305E40_aat* allele is now widespread. It was probably selected early in eggplant domestication, due to human preference for fruits with different tonalities, and spread in the development of different local varieties and accessions now ascribable as ‘Type 2’. For four accessions (Table 2), however, the HPLC analysis confirmed a discordance between the HRM haplotype and anthocyanin type: two samples displayed an alternative HRM profile (AATv) with respect to the allelic forms of ‘67/3’ and ‘305E40’, presumably reflecting the presence of an additional SNP variant in the HRM fragment, which affects the melt profile, whereas in two other samples the HRM profile confirmed a *SmelAAT* (AA) haplotype in fruits characterized by purple peel color (P) and a D3R HPLC profile, opening the possibility that another uncharacterized mutation is present in these accessions.

## 4. Materials and Methods

### 4.1. Plant Materials and Peel Samplings

*Solanum melongena* plants were grown in pots (30 cm diameter) in a glasshouse at CREA-GB (Montanaso Lombardo, Italy), under standard conditions. The ‘305E40’ is a double haploid line derived from an interspecific somatic hybrid *Solanum aethiopicum* gr. *gilo* (+) *S. melongena* cv. Dourga [45], which was repeatedly backcrossed with the recurrent lines ‘DR2’ and ‘Tal1/1’ prior to selfing and, finally, another culture [27]. The ‘67/3’ is an F8 line selected from the intraspecific cross cv. ‘Purpura’ × cv ‘CIN2’ [27]; ‘DR2’ is a breeding line utilized as a recurrent parent in some cycles of the breeding program, which yielded the line ‘305E40’. Three plants of each of the 61 accessions from the eggplant germplasm collection available at CREA (detailed in Table 2), selected as displaying a wide range of different anthocyanin peel colors, were grown under the same field conditions. The fruit color of all accessions was evaluated using at least two representative fruits per plant, collected at the commercial stage, and visually classified into Purple or Lilac, commonly considered as being associated to the exclusive presence of D3R or NAS, respectively. Samplings for molecular analyses were carried out using at least three fruits for each individual plant harvested at two fruit-ripening stages according to Mennella et al. [13]: unripe (stage A), approximately 21 DAF (days after flowering), and commercially mature (stage B), approximately 38 DAF. HPLC peel biochemical analyses were carried out on three peel samples collected from a single fruit for each plant. Peel samples were obtained from freshly harvested fruits of T1, untransformed control plants and selected accessions, by using surgical scalpel blades, avoiding as much as possible to collect flesh material. Each peel sample was constituted of about 1.0 g of skin taken from the equatorial part, immediately frozen in liquid nitrogen, and stored at −80 °C. The samples devoted for HPLC were freeze-dried.

### 4.2. Molecular Phylogenetic Analysis by Neighbor Joining Method

Peptide sequences from different species used for phylogenetic analysis were obtained through BLASTp by using the *SmelAAT* protein sequence to search the NCBI database (https://www.ncbi.nlm.nih.gov, accessed on 10 September 2019) to obtain *S. lycopersicum*, *S. tuberosum,* and *C. annuum* sequences; the Sol genomics database (https://solgenomics.net, accessed on 10 September 2019) for *Petunia axillaris*; and the eggplant genome database (http://www.eggplantgenome.org, accessed on 10 September 2019) for *S. melongena.* Sequences were chosen using e^−60^ as e-value cutoff. The *V. vinifera* sequence *XP_010648156* (identical to *Vv3AT* [46]) was also obtained from NCBI and used as an outgroup. Phylogenetic relationships were estimated in MEGAX [47]. Peptide sequences were aligned by MUSCLE with default settings. Evolutionary relationships among sequences were inferred by using the neighbor joining method based on the JTT matrix-based model. The rate variation model allowed for some sites to be evolutionarily invariable, and a discrete Gamma distribution was used to model evolutionary rate differences among sites. The reliability of the phylogenetic tree was estimated by setting 500 bootstrap replicates.

### 4.3. DNA Extraction and HRM Analysis

DNA samples were extracted from frozen (−80 °C) young leaves using the GenEluteTM Plant Genomic DNA Miniprep kit (Sigma, St. Louis, MO, USA). Genotyping was carried out via the High-Resolution Melting (HRM) technique [48] utilizing the EvaGreen supermix kit (Biorad, Hercules, CA, USA) and run using a Rotor-Gene 6000 (Corbett Research, Mortlake, NSW, Australia) PCR machine utilizing the HRM^TM^ with pre-Amplification program. Primers for HRM are detailed in Table S1. Both melting curve (range 50–80 °C with increment of 1 °C per cycle) and HRM (55–70 °C, increment of 0.1°C per cycle) functions were utilized for analysis of genotyping data. HRM haplotype was scored as “AA” for *SmelAAT*, “aa” for *305E40_aat* variant, and “Aa” for the heterozygous. Different peaks not attributable to any of the listed categories were considered as AAT variant (*AATv*).

### 4.4. Cloning of the SmelAAT cDNA Sequences

*The SmelAAT* (*Smel005g236240.1.01*) sequences were amplified by PCR, starting from total eggplant peel cDNA of the ‘67/3’ and ‘305E40’ lines, using the Phusion High-Fidelity DNA Polymerase (Thermo Fisher Scientific, Waltham, MA, USA). Primers for amplification and cloning are detailed in Table S1. The amplified genes were Sanger sequenced. Full length cDNA of *SmelAAT* from ‘67/3’ (GeneBank submission Id2424224) was cloned into pENTR/D-TOPO vector (Thermo Fisher Scientific, Waltham, MA, USA); the entry clones were recombined with destination vectors pK7WG2.0 containing the constitutive 35S promoter via Invitrogen TM Gateway TM recombination cloning technology (Thermo Fisher Scientific, Waltham, MA, USA). The binary vector pK7WG2 containing a NPTII selection cassette flanked by T-DNA border sequences containing the *p35S:SmelAAT* was used to transform *Agrobacterium tumefaciens* strain GV2260.

### 4.5. RNA Extraction and RT-qPCR Analysis

Total RNA from eggplant peel samples was extracted using the TRIzol RNA Isolation Reagents (Thermo Fisher Scientific, Waltham, MA, USA) combined with the Spectrum Plant Total RNA kit (Sigma Aldrich) and treated with the RQ1 RNase-Free DNase Kit (Promega, Madison, WI, USA). The single-strand cDNA was synthesized from 1 μg of RNA using the ImProm-II™ Reverse Transcription System Kit (Promega, Madison, WI, USA). A mixture of 0.5 μg/reaction of Oligo(dT) and 0.5 μg/reaction of random primers was used for first-strand synthesis. The reverse transcription quantitative polymerase chain reactions (RT-qPCR) were carried out according to the following PCR parameters: 95 °C for 5 min, followed by incubation for 15 s at 95 °C, denaturation for 15 s at 95 °C, and annealing for 60 s at 59 °C for 40 cycles, followed by elongation at 72 °C for 20 s. The reaction was performed using GoTaq^®^ RT-qPCR Master Mix by Promega, Madison, WI, USA. The reaction contained 1.0 µL of previously diluted cDNA (1:20), from 0.2 µL to 1.0 µL of primers (1 µM each), 5 µL of GoTaq^®^ RT-qPCR Master Mix, and RNase-Free water up to the final volume of 10 µL. All reactions were performed in triplicate with three biological replicates, and no-template controls were included in all analyses. Standard curves for each primer pair were calculated across a 5-fold dilution series of pooled, diluted cDNA amplified in technical triplicate. Primers for RT analysis of *SmelAAT* and *Smel5GT1* were designed based on the available genome sequence of ‘67/3’, utilizing the Primer 3 software (https://bioinfo.ut.ee/primer3-0.4.0/primer3/, accessed on 10 February 2020).

The primers designed to amplify all the AAT transcripts were: *SmelAAT* in ‘67/3’, *305E40_aat* in ‘305E40’, and ‘DR2’, *305E40_aat* + *p35S::SmelAAT* in transgenic plants #18-1, #49-1, and #32-2.

For expression analysis of the transformed plants, two additional reverse primers were designed to distinguish the expression of the endogenous gene (either *SmelAAT* or *305E40_aat*) from the *p35S::SmelAAT* transcript. Specific reverse primers were designed on the *3’UTR* region of the endogenous gene or those of the *p35S::SmelAAT* cassette, respectively. All primers are detailed in Table S1. The PCR efficiency was calculated by Rotor-Gene 6000 Series Software, and it was optimized to be in the range of 90%–100% with R2 values of 0.996. Specificity of amplifications was assessed first by PCR for the presence of a single band and then through the melt curves’ analysis. The relative expression levels of the target genes were normalized, as suggested by Bustin et al. [49], by using the two reference genes *SmelGADPH* (Glyceraldehyde 3-phosphate dehydrogenase) and *Smel18S* [50]. The relative quantification of gene expression was performed using the geometric averaging method [51].

### 4.6. Eggplant Transformation

The procedure for transformation of eggplant cotyledon from in vitro grown ‘305E40’ and ‘DR2’ plantlets was essentially as described previously [52]. Media for the 2 days pre-culture and selection were as in Arpaia et al. [53]. For explants’ infection, overnight GV2260 *A. tumefaciens* liquid culture was centrifuged, and the pellet resuspended at 0.1 OD600 in MS basal medium, 2% glucose, and 200 mM acetosyringone pH 5.5. All the explants were infected by dipping in bacteria suspension for 5 min, blotted dry onto sterile filter paper, and placed back into the pre-culture medium and kept in the dark. After 48 h, the explants were transferred to selective medium without acetosyringone and supplemented with 30 mg/L kanamycin and 500 mg/L cefotaxime. The explants were subcultured every 3 weeks onto selective medium. Calli with compact green nodules were then transferred to a regeneration medium [53]. Regenerated shoots were rooted and propagated in V3 medium [54] without antibiotics. Plants’ material for transformation, calli and transformed sprouts, were grown in in vitro conditions in a growth room chamber with 16 h of light by means of fluorescent lamps at ~50 µE m^−2^ s^−1^ intensity at 25 ± 2 °C. Putative kanamycin-resistant transgenic plantlets were confirmed by PCR for the presence of the insert, using the primers NPTII fw and NPTII rev (Table S1). The PCR-positive, transformed T0 plants, after ex vitro adaptation, were potted and grown under glasshouse condition for phenotypic-genotypic evaluation and self-pollinated. The T1 progenies were sown in plastic trays of 104 holes, and seedlings at two leaves’ stages were sprayed with a 200 mg/L kanamycin, according to Sunseri et al. [55]. The resistant non-chlorotic T1 plantlets were checked by PCR for the presence of the transgene. At least three Kan^R^ T1 plants from each T0 event were grown until fruiting, and one of them was exclusively employed for HPLC and RT-qPCR analyses.

### 4.7. Anthocyanins’ Extractions and HPLC Analytical Conditions

The freeze-dried peel samples were powdered and stored at −80 °C. The anthocyanins’ extraction was carried out through a double extraction, according to the rationale of Ichiyanagi et al. [22]. A first extraction was performed on 100 mg of lyophilized and powdered peel using 2.0 mL of methanol acidified with 0.1% TFA. Then, the samples were sonicated for 5 min at room temperature and centrifuged at 4 °C at 10,000× *g* for 10 min. After centrifugation and collection of the supernatant, the pelleted tissue sample was submitted to a second extraction with 1.5 mL of acidified methanol. The two extracts were combined, filtered through a 0.2-μm PTFE syringe filter. Filtered extracts were stored in capped, brown-glass vials at −80°C until being removed for High Performance Liquid Chromatography (HPLC) analysis. An RP-HPLC-DAD method (Jasco-Europe, Cremella, Italy) was developed to separate the extracts of the eggplant peel, following the method used in the work of Braga et al. [56], with some modifications. Anthocyanins were identified by their UV-VIS spectra (525 nm was selected for chromatography) and quantified by a calibration with external standards, made with purified crystals of D3R and NAS [57], respectively. The used analytical column was an Inertsil ODS-3 (GL Sciences, Tokyo, Japan), 250 × 6 mm i.d., kept at 45 °C. A gradient elution was performed, using as mobile phase, with solvent A as formic acid 0.5% in water and solvent B constituted by formic acid 0.5% in acetonitrile. The flow rate was 0.7 mL/min. The gradient was started for 20 min at 98% solvent A, then was led to 50% solvent A in 15 min, left at 50% solvent A for 10 min, and returned to 98% solvent A in 10 min. Samples of 20 μL were injected and monitored by the DAD system at 525 nm. In these conditions, retention times of D3R and NAS were 19.3 and 23.2 min, respectively. The amount of anthocyanins was expressed as mmol/kg (mM) of peel dry weight.

### 4.8. Statistical Analysis

Statistical analysis was performed using the JASP Team (2020) software (Version 0.14.1.https://jasp-stats.org/) and graphs were realized using GraphPad Prism 9. One-way ANOVA with three biological replications was employed. Tukey’s HSD (*p* < 0.05) post hoc test was performed for means comparison. The correlation (R) was calculated by using the Pearson correlation coefficient and the Spearman’s rank correlation. The coefficient of determination (R^2^) was used to estimate the causal relationships between the variability of the dependent factors.

## 5. Conclusions

In this work, we provide evidence for the crucial role of *SmelAAT* encoding gene in the final decoration of anthocyanins in eggplant peel, as a single nucleotide deletion is responsible for the difference between D3R or NAS type in several cultivated eggplants. Finally, an indel marker strongly predictive of the type of accumulated anthocyanin was developed and will be a powerful tool for marker-assisted selection in breeding programs.

## Figures and Tables

**Table 1:** Concentration in the peel (mM, dry matter) of D3R and NAS, and percentage of NAS over the total (D3R+NAS) amount of anthocyanins in fruits of lines ‘67/3’, ‘DR2’, and ‘305E40’, and 12 independent T1 plants transformed with *p35S::SmelAAT*.

Genotype		D3R	±sd	NAS	±sd	NAS
		mM		mM		%
DR2	Ctr	37.41	4.00 ^b^	0.00	0.00 ^f^	0
DR2	14-1	8.65	0.75 ^ef^	0.29	0.42 ^f^	2.91
DR2	18-1 *	9.12	0.64 ^ef^	7.46	0.45 ^b^	45.03
DR2	3-1 *	43.96	1.28 ^a^	3.20	0.05 ^de^	6.78
DR2	35-1	20.94	0.25 ^c^	5.88	0.14 ^c^	21.91
DR2	49-1 *	14.66	1.84 ^d^	2.18	0.27 ^e^	12.93
DR2	5-5	6.74	0.15 ^fg^	3.88	0.32 ^d^	36.52
DR2	51-1	3.95	0.17 ^gh^	0.04	0.03 ^f^	0.93
DR2	6-3	1.38	0.16 ^h^	0.11	0.02 ^f^	7.61
DR2	8-1 *	12.63	1.22 ^de^	3.14	0.51 ^de^	19.85
DR2	8-2	25.12	0.70 ^c^	5.22	0.34 ^c^	17.19
DR2	32-2 *	23.32	1.59 ^c^	9.18	1.02 ^a^	28.21
305E40	3-2	9.07	0.24	2.97	0.03	24.67
305E40 ^++^	Ctr	12.69	0.27	0	0	0
67/3 ^++^	Ctr	0	0	3.28	0.05	100

Ctr, untransformed control; * analyzed by RT-qPCR. ^++^ Data gathered from Toppino et al. [24]. For each column at least a common letter indicates no significant difference among the ‘DR2’ genotypes including overexpressing T1 and ‘DR2’ Ctr plants (Tukey test, *p* < 0.05).

**Table 2:** Validation of the HRM_AAT marker in different anthocyanin colored CREA accessions.

Name	Code	HRM Genotype	Peel Visual Color Phenotype	HPLC Phenotype
DADALI	AM 001	AA	L	-
CIMA VIOLA	AM 004	aa	P	-
1F5(9)	AM 010	aa	P	-
CCR3	AM 013	aa	P	-
VIOLETTA SAIS	AM 014	AA	L	-
LUGA 063	AM 015	aa	P	-
PROSPEROSA	AM 016	AA	L	-
LUNGA VIOLETTA	AM 018	AA	P *	NAS
TAL1/1	AM 021	aa	P	-
ANGIO	AM 022	AA	L	-
DR2	AM 026	aa	P	-
FANT E13D	AM 029	AA	P *	D3R *
SNL 600	AM 034	AA	L	-
CIN 01/24-6	AM 035	AA	P *	D3R *
VIOLA CIN	AM 036	AA	L	-
V. TOSCANA	AM 037	AA	L	-
44074	AM 042	AA	L	-
55-08	AM 045	AA	L	-
16-09	AM 046	AA	L	-
P621-08 (74-4)	AM 047	AA	L	-
P328	AM 053	AATv	P	D3R *
S1052-08	AM 056	AATv	P	D3R *
LS 3805 MINDEN	AM 086	AA	L	-
LS611	AM 103	aa	P	-
NAGA UNGU	AM 106	AA	L	-
N286	AM 107	aa	P	-
N24	AM 110	aa	P	-
N243	AM 111	aa	P	-
N286	AM 112	aa	P	-
N321-14	AM 113	Aa	L	NAS
PI17	AM 124	aa	P	-
LUNGA MARINA	AM 139	aa	P	-
BUIA	AM 156	aa	P	-
ANK2	AM 158	aa	P	-
ANGIO3	AM 167	AA	L	-
SM 19/14	AM 170	aa	P	-
PALERMITANA	AM 171	AA	L	-
JM	AM 174	AA	L	-
THAI TH472	AM 179	aa	P	-
LISTADA	AM 180	aa	P	-
THAI TH449	AM 190	aa	L *	D3R
THAI TH4760	AM 199	aa	P	--
TOPAK	AM 217	aa	P	--
PI169648	AM 221	aa	P	-
TOPATAN	AM 222	aa	P	-
PI171859	AM 232	aa	P	-
L129	AM 268	AA	L	-
DRS4	AM 274	aa	P	-
CAAS 6	AM 280	AA	L	-
CAAS 16	AM 290	AA	L	-
CAAS 17	AM 291	AA	P *	NAS
LONGO	AM 300	aa	P	-
TALINDO purple	AM 302	aa	P	-
BANGLADESH	AM 318	aa	P	-
USTICA	AM 322	AA	L	-
INDIA1	AM 360	AA	L	-
L316	AM 371	aa	P	-
LP742	AM 378	AA	L	-
L. VIOLA MEDIA	-	aa	P	-
ANOMINORI	-	AA	L	-
VIOLA OVALE	-	AA	L	-

For each accession: common name, accession code, HRM haplotype (*SmelAAT* = A, *305E40_aat* = a, *AAT* unknown variant = AATv), peel color (Lilac = L, Purple = P), and HPLC phenotype (nasunin = NAS, delphinidin-3-rhutinoside = D3R). Accessions with no correspondence between genotype and phenotype (either visual and/or HPLC) are marked with (*).

## Data Availability

Data are contained within the article. Full length cDNA of *SmelAAT* from ‘67/3’ was submitted to GeneBank and is available with reference code Id2424224.

## Supplementary Materials

The following are available online at https://www.mdpi.com/article/10.3390/ijms22179174/s1.
