# Supplementary material for: A SmelAAT Acyltransferase Variant Causes a Major Difference in Eggplant (Solanum melongena L.) Peel Anthocyanin Composition
Source: Int J Mol Sci. 2021 Aug 25;22(17):9174. doi: 10.3390/ijms22179174 (PMC8431300; doi:10.3390/ijms22179174)
Supplement: Supplementary file 1 [file ijms-22-09174-s001.zip › ijms-1321836-supplementary.pdf]

Supplementary Material

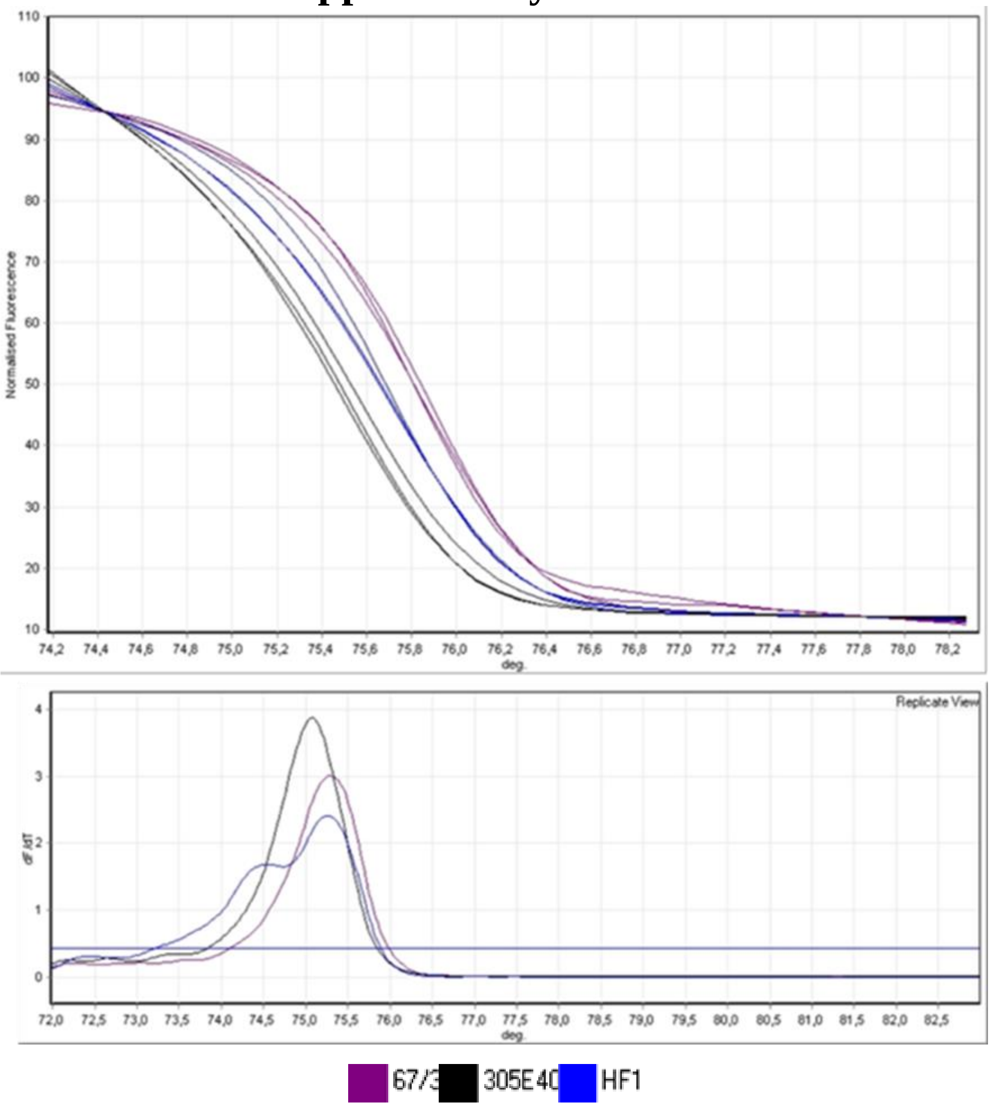

Figure S1. Output of Rotor-Gene 6000 Series Software 1.7 for the Melting curve of the AAT_HRM Marker.

Table S1: List of primers used.

Primers Name	Sequences 5'-3'	Use
Seq_SmelAAT_FW	tttatacttccctgggagttg	Sequencing
Seq_SmelAAT_RV	aataaggtacgctcccagaag	Sequencing
HRM_AAT_FW	atgagccaaattacaaaacaaaacttaaatg	HRM
HRM_AAT_RV	aggcacagcaaaattaggagc	HRM
GW_SmelAAT_FW	caccatgagccaaattacaaaacaaaa	Gateway cloning
GW_SmelAAT_RV	tagaaattccaaaatctttcaag	Gateway cloning
NPTII_FW	tgctcctgccgagaaagtat	PCR
NPTII_RV	agaactcgtcaagaaggcgatag	PCR
qPCR_SmelAAT FW	cgtgcaaatggtccacacat	RT-qPCR
qPCR_SmelAAT RV	ttccccttccccatacgact	RT-qPCR
qPCR_Smel5GT1_FW	tccattcaacttcctggcct	RT-qPCR
qPCR_Smel5GT1_RV	aggcttccttttgcacttga	RT-qPCR
qPCR_SmelAAT_3′UTR FW	attgaagctagagtttgctt	RT-qPCR
qPCR_SmelAAT_3′UTR_RV	aataaggtacgctcccagaa	RT-qPCR
qPCR_SmelAAT_35S:3′UTR_RV	gctgaacttgtggccgttta	RT-qPCR
qPCR_SmGAPDH_FW	ggtgccaagaaggttgtgat	RT-qPCR
qPCR_SmGAPDH_RV	ccaatgctagttgcacaacg	RT-qPCR
qPCR_Sm18S_FW	atgataactcgacggatcgc	RT-qPCR
qPCR_Sm18S_RV	aaacggctaccacatccaag	RT-qPCR

Table S2: F2 population from the cross 305E40 × 67/3. For each F2 individuals: Individual number, HRM haplotype (SmelAAT=AA, 305E40_aat=aa, Heterozygous=Aa), peel colour (lilac=L, purple=P), HPLC phenotype (nasunin=NAS, delphinidin-3-rutinoside =D3R). The individuals where marker and phenotype (colour and/or HPLC) do not match are marked (*). The statistical analysis was performed by using the Spearman's rank correlation.

Individual	HRM Haplotype	Peel Colour Phenotype	HPLC Phenotype
**Parental**			
67/3	AA	L	NAS
305E40	aa	P	D3R
HF1	Aa	L	NAS
**F2 Population**			
1	aa	P	D3R
2	Aa	L	NAS
3	Aa	L	NAS
4	aa	P	D3R
5	Aa	L	NAS
6	Aa	L	NAS
7	Aa	L	NAS
8	aa	P	D3R
11	Aa	L	NAS
12	Aa	L	NAS
13	AA	L	NAS
14	Aa	L	NAS
15	aa	P	D3R
16	Aa	L	NAS
17	aa	P	D3R
19	aa	P	D3R
20	Aa	L	NAS
21	Aa	L	NAS
22	AA	L	NAS
23	aa	P	D3R
24	AA	L	NAS
25	AA	L	NAS
26	AA	L	NAS
27	AA	L	NAS
28	AA	P*	NAS
29	Aa	L	NAS
30	AA	L	NAS
31	Aa	L	NAS
32	Aa	L	NAS
33	Aa	L	NAS
34	Aa	L	NAS
35	AA	L	NAS
36	AA	L	NAS
37	AA	L	NAS
38	Aa	L	NAS
39	Aa	L	NAS
40	AA	L	NAS
41	AA	L	NAS
42	Aa	L	NAS
43	Aa	L	NAS
44	AA	L	NAS
45	Aa	L	NAS
46	Aa	L	NAS
47	Aa	L	NAS
48	Aa	L	NAS
49	Aa	L	NAS
50	aa	P	D3R
51	Aa	P*	NAS

52	AA	L	NAS
53	Aa	L	NAS
54	AA	L	NAS
55	AA	L	NAS
56	AA	L	NAS
57	Aa	L	NAS
58	AA	L	NAS
59	Aa	L	NAS
60	AA	L	NAS
61	AA	L	NAS
62	Aa	L	NAS
63	AA	L	NAS
64	aa	P	D3R
65	AA	P*	NAS
66	aa	P	D3R
67	Aa	L	NAS
68	aa	P	D3R
69	AA	L	NAS
70	aa	P	D3R
71	Aa	L	NAS
72	aa	L*	D3R
73	Aa	L	NAS
74	AA	L	NAS
75	AA	L	NAS
76	AA	L	NAS
78	Aa	L	NAS
79	AA	L	NAS
80	Aa	L	NAS
81	Aa	L	NAS
83	AA	L	NAS
84	aa	P	D3R
85	AA	L	NAS
86	Aa	L	NAS
88	aa	L*	D3R
90	aa	L*	D3R
91	aa	P	D3R

File S1: Sequences from NCBI and Sol Genomics databases used to identify putative acyltransferase amino acid sequences from S. melongena, S. lycopersicum, S. tuberosum, Capsicum annuum and Petunia axillaris used to obtain a molecular phylogenetic tree.

>*Eggplant_SMEL_005g236240*

MSQITKQNLNGTFIELEILNEKLIKPSLPTPNHLNSYKLSFFDQIAPNFAVPLLYFYPPVPPEKLNLQHAEEVHKQLQNSLSDVLTKF

YPLAGRLSEDGTSIECHDQGVIYLEAKVNCQLNEFLDKAYKDSDLVKIFVPPIRIRLAELPNRPIMAIQATMFEHGGLALAVQMVH

TLGDGFSGCAITDEWAKVSRMGKGNTRNLQFRSDLAKIFPPKDNIFEMIKKGRPRGYEMKIATRIFIFDEIAISKLKENVNKSLSYY

SSRVEVLTALIWRSLMRLRQGHNRPSMLQFAINLRGRGSPKLLGEDQNFFGNFYLDIPIKCVPSQSNQDPELHEIVTLIRNAKNKIL

SDIANASSEEIFSILIESLNQIREGYNDDEIDLYPTSSLCRFPLNESDFGWAKPIWVSRVNVPFQMFFLMDAKDGIEARVCLNGDD

MIKLEKDVDIVEFSYVPK

>*Eggplant_SMEL_001g121710*

MAKSDIQLQMRKMIRPSTPTPNHLRSLKLSFFDQGAPRIYVPIMFHYLPTSEWNREVVTEKLQNSLAETLTNFYPLAGRFREDEFS

IHCNDEGVEYVETKVVNSDLIEFLQHGPKNIELLNDLLPDMDDHRPSSPLLGVQVNVFNCGGIVIGIQISHMVADAFTLATFVNE

WAHTSLTGTTKDCLPSFGHLSSLFPPRELSGPQFSPPSNTGPKIITRMFVFDVLAIAKLKDRINSSGTLTRPTRVVVVMSLIWKVLM

GISSAKHGHPRDSSFLFPINLRGKSKLSSLGHAQGNFYVSVAATLEANESRRELNEFVNAIGITARNTSLSIGNASVDKIISLSINCGT

EIIKKFGQGDEIDIYASTSWCRFPWYEADFGWGKPIWVSSVGRTFEVISLFDTKNGDGIEAWVSLKENDMIEFERDPHILSSTSKEN

VKALNSHPKSSWEPQAFVVQSAGSSPVLFHYLPSTSMTQVTITERRQKSLVEKLTKLYPLAGRIREDEFSIHCNDEGVEYVEAKVV

NVDLAEFLHQGPKNIELLNDHQPSSPLLGVQVNVFRCGGVVIGIKISHMIADAFTLATFVNEWAHTSLTGTPKQSHSLLSFSHLSLL

FPTRLLSGPQFSAPAHNNKIVTRRFVVDVSAIANLRSTIKDSRLTRVVVVMSLIWKVLTGISTAKHGHSRDSCLSFPVNLRGKSNIPS

LEHALGNCATIGIVTNEARTELNEFVNLVGKTIGDICEGIGAASFDDITSMCITCVTEIKQIWSKRRRQLFQHELV

>*Eggplant_SMEL_010g340140*

MKDPMQIKVLSKHLIKPSSPTPNNLKHYKLSFFDQVADVAHLPLVLFYPYCDKNSKNEELEESLSRILTHVYPLAGRFAEDESSVLC

LDQGAMFIKATVNCKLDDFLQQAKKDNDLALSFWPHGIMDVDETNLFLSPLLVVQVTTFECGGLALAISNAHPATDGFTAFKIV

YEWAKVCKLGTPSKEINFMNFNLGTLFPSKDLSTLLEPPIDEGKRIESKLIARKFVFDEDAILRLREKFDSTGEGLGFKPSRVEMITSL

LWRSLIRAARSPDLKRSIMSFPLNLRGKVEAFPEIVNSFGNLIIEIPIKFEHNDEIEIESLHHVVKLIRESVQATIHKCAKATSDEIVSLV

IDLYKDSYAGLEWGGDNEVVNFTCSSLCRFPVQKTDFGWGKPSLMHFGSRHSQIFWLYDTECETGIAVQMDLEEKYMDKLVRD

KDIMDLAKF

>*Eggplant_SMEL_005g240350*

MELNLEFISTKLIKPSLPTPQHLKNYKLSFFDQLAEREHMPLLLFYGSNNNNSIDDDKFDEKLEKSLSRILCHVYPAAGRLSRDGCS

IDCLDQGVTFTKAKVNCQFDDFINQVRNDLNLALFFFPKEIQDLKDAEFDTTPPVVVQVTEFQCGGIAISISASHPVMDGFTNFKF

VYEWAKVCKLGIPAEEIDFLSYDFCEILPARDLSSTFPNRVHPEYLGAKFIAKRFVIDELTISQLRNKLARAMDSGELCFKPSRVEIIT

AILWRALIRVSEAKHGYLRRSLVFFPVNLRGKISLPLKENAFGNYVMDAPIKFEPGKNKMELHDFVTLIRNSVQKAIDACATGSAD

DIVAKVADTYKEIFVSKEWGTDNDEVDKCLISSLCKFPMQEADFGRGKPSLMHFGLRNFDSCWMYDGECGSICVQVDLKDTYM

RLFECDDDIKTFTRLY

>Eggplant_SMEL_005g240370

MAFHKENMKVEITSTKFIKPSSPTPNHLQNYELSFFDQITELQHLPFVLFYSPPTTNNTHSYASYEEQFEQSLSRILTHVYPIAGRFV

DEGSISCQDQGVKFVKAKVNNKLNEFLEEVHKDVNLALICWPQEAWAADESNLFTFPIVIVQITEFACGGVALSVSHVHSVMDGY

SMFNIINEWSKVCRLDIPVEKIDFLSFNLADVFPSRDLSKLLLPRIPGEDGMDEKLVAKRLYINEDSISRLREKVKDLCFNPSRVEIIIA

LLWRALIRASEKKHEYLRRSLMGVPINFRAKLTSLSHEVQKSFGNLVMDTPVKFVPGHGDNHQIPELHEFVKLIRDSVKETISSVDK

SSPEDVVHAVANICNENFVSPEWGGNKEVDVYGCSSLCRFPIQEADFGWGKPCLLHFGSRRGQYCWLYDAECGNGICVQVDLKE

DNMHLFECDTDIKDFFEF

>Eggplant_SMEL_010g340130

MKDPMQIKVLSKHLIKPSSPTPNNLKHYKLSFFDQVADVAHMPLVLFYPHCDKNSKNEELEESLSRILTHVYPLAGRFTEDESSILC

LDEGVTFIKATVNCKLDDFLPQAKKDHDLSLSFWPHGIMDVDETNLFVTPLLVVQVTTFECGGLALAVSTAHPATDAFTAFKIIYE

WAKVCKFGTPSKEINFMNFNLGTLFPSKDLSTLLEPPVDEGKRIESKLIARNIVFDEDAISRLTKKFEHSTSEGLTFKPSRVEMITALL

WRSLIRAAKSPDLKRSVMAFPVSLRGKVEAFPETINSFGNLIIEIPIKFEHNDETKIESLHHIVKLIRQTVQEIINKCAKATPDEIAALVI

DLYNDGYGGFIWGGDREVVNFTCSSFRRFPVQNTDFGWGKPSLMHFGSRHSQVFWLYDTECETGIAVQMDLEEEYMDKFVHD

KDIMDFAKF

>Eggplant_SMEL_005g240520

MAFQKENMKVEIISTKFIKPSSPTPNHLQNYKLSFFDQITDLDFLPVVLFYPPPTNNTSTYASYEEKIEQSLSKIITHVYPIAGRFIDEE

SISCQDQGVKFVKAKVNNKLNEFLEEVHKDVNLALICWPQEAWAADESNLFTFPIVIVQITEFACGGVALSVSHVHSVMDGYSMF

NIINEWSKVCRLDIPVEKIDFMTLNLAHVFPSRDLSQLLLPRIPLEYGMNEKFVAKKLYINEDSVSRLRERVKGLSFNPSRVEIITALL

WRALIGASQKKHGYLRRSLMCVPVNFRTKLISLPHEVEKSFGNLVMDAPVKFVPGHGGNYQIPELHEFVKLIRDSVKETISSVDKS

SPEDVVHAVANICNENFVSPEWGGNKEVDVYGCSSLCRFPIQEVDFGWGRPCLLHFGSRHAQYCWLYDAESGYGICVQVDLKE

DNMHLFECDTDIKDFFEF

>Eggplant_SMEL_005g240420

MKVEIISTKFIKPSSPTPNHLQNYKLSFFDQITDLDFLPVVLFYPPPTNNTSTHASYEEQFEQSLSKIITHVYPIAGRFIDEESISCQDQ

GVKFVKAKANNKLNEFLEEVHKDVNLALICWPQEAWAADESNLFTLPIVIVQITEFACGGVALSVSAVHTAMDAYSIFNIINEWSK

VCRLDIPVEKIDFMTLNLAHVFPSRDLSQLLLPRIPLEYGMDEKFVSKRLYINEDSISRFRERVKGLSFNPSRVEIITALLWRALIGAS

QKKHGYLRRSLMCVPINFRTKLISLPNEVEKSFGNLVMDAPVKFVPGHGDNYQIPELHEFVKLIRDSVKETISSVDKTSPEDVVHA

VANICNENFVSPEWGGNKEVDKYGCSSLCRFPIQEADFGWGKPCLLHFGSRSDQYCWLYDAECGNGICVQVDLKEDSMHLFEC

DTDIKDFFEF

>Eggplant_SMEL_010g340090

MKDSMQVKILSKSFIKPSSPTPDHLKIHKLSFFDQVADIAHLPLVLFYPHCNNNSKTNEQLEDSLSKILSYFYPLAGRFTEDESSVLC

IDQGVTYIKATVDCKLDDFLQHANMDLDLVLSFWPHGIMDVDETNLFVTPLVVVQVTTFECGGTAVGFSCAHPAIDGFTAFTFIY

EWAKLCKFGNPSKEVNFMSFNLGTTIFPARDLTNLLQSPVDEGKRTGSKLIGRKLIFDEAALSRLAKKFDSGNVALSFKPSRVEMIT

AFLWRSLIRAAGAGNPHLKRSVMAFPFNLRGKVAAFPETANSFGNLIIEIPIKLEHNDETETESLHQIVKLIRETVQETICRCAKATP

DEMVSLVLDLYNEYYGGLKWGGDREVVNFTCSSLCRFPIHKADFGWGKPSLMHFGSRHSQVFWLYDTECETGIAVQMDLEQTY

MNNLLRDQDIMDFAKF

>Eggplant_SMEL_005g240400

MAFQKENMKVEITSTKFIKPSSPTPNHLQNYKLSFFDQITDLELLPFVLFYPPPTNNTNTYASYEEKFEKSLSKIITHVYPIAGRFIDE

ESISCQDQGVKFVKAKVNNKLKEFLEEVHKDVNLALICWPQEAWAADESNLFTFPIVIVQITEFACGGVALSVSHVHSVMDGYSM

FNLVNEWSKVCRLDIPVEKINFLSFNLADVFPSRDLSKLLLPRIPEEDGMDEKSVAKRLYINEDSISRLREKVKGLSFNPSRVEIITAL

LWRVLIRASQKKHGYLRRSLMCVPINFRSKLISLSHEVEKSFGNLVMDAPVKFVPGHGDNYQIPELHEFVKLIRDSVKETIVSVDKT

SPEDVVHAVANICNENFVSPEWGGNKEVDKYGCSSLCRFPIQEADFGWGKPCLLHFGSRSDQYCWLYDAECGNGICVQVDLKE

DSMHLFECDTDIKDFFEF

>Eggplant_SMEL_005g240450

MAFQKENMKVEIISTKFIKPSSPTPNHLQNYKLSFFDQINDETHLPLVLFYPPTNNFNFAAHEEQFEQSLSRILTHVYPIAGRFIEGN

SISCQDQGVKYVKAKVNSKLNGFLGKAHKDVSLALLCWPQDTCIVDESNLFTSPIVIVQITEFKCGGVALTVSHVQTAMDGYSTLT

VISEWSKVCRLNIPAEKIDFMSSFNLAHVFPLGDLSKLLLPWVPEEDHMDAKLVAKRLYINEDSISRLRQEVGDLCFKPSRVEMIIA

LLWRALIRASEKKHGYLRRSLMAVPINLRTKLISLPHEVEKSFGNLVIDAPVKFIPGHGDNYKIPGLHEFVKLIHDTVKETIINCDKTS

PEDVVRALANIYNESFLAQEWGGNKEVDKYECSSLCRFPIQEADFGWGKPCLMHIGSRHAQYCWLYDAECGNGICVQVDLEEIN

MHLFESDSDVKVFFEF

>Eggplant_SMEL_001g121740

MAKLEIEIQTRKMLKPSSPTPNHLRILKLSLFDQMASRLHVPILFHYLPTSEEGIIAETCDKLQKSLGETLTKFYPLAGRFREDELSIH

CNDEGVEYVETEVNADLAEFLHQEPKTELLNDLLPEMDHTSSPLLGIQVNLFNCGGIVMGIQISHILADGFTLGTFVKEWTSISQT

GTAKDCLPSFGHLPSLFPSRVLSGPYILPSSDRGTKIVTRRFVFDVSAIARLKERINSSAMLPRPTRVVVVMSLIWKVLTGISTAKHG

HSRDSHLLFPVNLRGKSNLPSVEHALGNFCHAGIATLEANQSRMELTDYVDRVGSTARDTSAFIAKASIDDITSMFVNYKMPCEK

MDMYFCTSWCRFPWYEADFGWGKPFWVSDVSKPAELITLIDTKSGDGIEAWIGLKEKDMAVFERDSDILTFCPPQN

>Eggplant_SMEL_005g240550

MDFSSQIEVKIMSKKLIKPSSPTPNHLQNYKLSFFDQLAEQAHLPFVLFYPKNNSKITTNNMIQQLEQSLSRMLTHVYPAAGRFDE

NKSSIICDDQGVTLIKAKVNRRMDDEFLQQAHNNLDIVMEFWPQGSKEVNASNVFMTPVMLVQITIFQCGGIALSTSTAHPAIDG

WTNFTFIYEWSKVCKSEIPAEKIDFMRFDLAKIFGPKDNMTFCDQAKPLEAKLVGENQIIDEVSLSKLREKLTNSGALGFTPSRVEM

VTAILWRGLLRASQAITGEMKSSVMSFPLNLRGKLINYREATNPFGNFIIDIPITYEPKRTTNMELQDFIVLIREAMQKTLDYCSETS

SPNEVIEMVANLYNKYYEGKQWGANEDVEEFTCSSLTTFRIQEANFGWGNPSLMHFGSRNNQVFWLYSTQCGKSIGVQMDLKE

KYMDFIQHDQEFLAFTKV

>Eggplant_SMEL_007g291610

MEKIENCKPDNVVVDCWRKKRNRFYPLAGRYRKDDFSIECNDEGVEYVETKVNADLAEFIHQAPENEVVDDLLAWRVPPKVDL

SSSPLLGIQVNRFKCGGLVMGIQSSHILVDAYTLGSFVNEWAHITKTGTTKACLPSFGQLASLLPARVQQGPQLSLVSNKGAKSVT

RTFVFDAWAITELKDKINSSSATMVKSTRVMAITSLIWKILTGISSAKHGHSRDSTLLFTMNLRGKTKLPSTEHALGNFFLLGIATLE

ANQSRKELHDFANAVRSTSRDTFSYIGKASIEDITSIFAKGFPLGQKDDMDFYVCSSVCRFPLYEADFGWGKPSWVSTNSKDMEFI

SLFDTKNGDGIEAWVSLTENDMAEFEKDPDILTYCQAQK

>Tomato_NP_001234419

MSQITTQNLNGTCIQIEILNEKLIKPSLPTPNHLNSYKLSFFDQIAPNFAVPLLYFYPPVPPENSHLQRVEEVHKQLQNSLSEVLTKF

YPLAGRLSEDGTSIECHDQGVIYLEAKVNCQLNEFLDKAYKDSDLVKIFVPPIRIRLAELPNRPMMAIQATMFEHGGLALAVQIVH

TTGDGFSGCAITDEWAKVSRMEKGNVRNLQFRSDLVEVFPPRDNILEMIKKGRPRGYEMKIATRIFMFDEIAISKLKENVNKFMS

YSSRVEVVTALIWRSLMRVVRLRHGHNRPSMLQFAINLRGRGSPRVVGEDQNFFGNFYLDIPIKYVSSRSNQDPELHEIVTLIRNA

KNKILSEIANASSEEIFSILIESLNQIREGYNDDEIDLYPTSSLCKFPLNESDFGWAKPIWVSRVNVPFQMFFLMDSKNGIEARVCLN

EEDMMKLEKDVDIVEFSYVPK

>Tomato_XP_004253033

MAFQKENMEIEIISTKFIKPSSPTPNHLQTYKLSFFDQVSDETHLPLVFFYPPTNNINFSSHHEEQLEQSLSRILTHVYPISGRFNEDI

NSISCQDQGVKFIKAKMNSKLNEFLDKAHKDVNLSLLCWPQDSWNVDPSNLFTMPLVIIQITEFECGGLALSMSHMHMTMDGY

STFSFINEWSKVCRHKIPLEKIDFMSFDLANVFPTRDLSKLLLPRIPPVDRVECKLVARRLYINEDSISRLREKVSGDLCKFKPSRVEM

IMAILWRAVIRASEKKHGYLRRSLMNIPINLRTRLISLPQVEKSFGNLGVDAPIKFIPEENKMELHEFVTLIHNAVKETITTCDKTSPE

DIVSAVSNIYNESFLAQDWGGNDEVDRIISSSLCKFPIQEADFGWGKPCLMHFGSRHGQVCWLYDAECGNGICVQVDLKEDNM

NLFECDNDIKDFFQF

>Tomato_XP_004253030

MAFQKENMQVEIISTKFIKPSSPTPNHLQIYKLCFFDQVTDETHLPLVLFYPPTNNINLSSHHEEQLEQSLSRILTHVYPISGRFNEDI

NSISCQDQGVKFIKAKMNSKLNEFLDKAHKDVNLSLLCWPQDSWNVDPSNLFAMPLVIIQITEFECGGLALSLSHVHMAMDGYS

TFSFINEWSKVCRLEIPVEKIDFMSFDLANVFPTRDLSKLLLPRVPTEDRVESKLVAKRLYINEDSISRLREKVGGDLCKFKPSRVEMI

TALLLRALIRASEKKHGYLRRSLMNIPINLRTRLTCLPQVEKSFGNLGVDAPIKFIPGENKMELHEFVTLIHNTVKETIATCDKTSPE

DIVFAVSNIYNKSFLAQDWGGSDEVDKYTSSSLCKFPIQEADFGWGKPCLMHFGSRHDQCCWLYDAECGNGICVQVDLKEDHM

HLFECDNDIKYFFSF

>Tomato_XP_004233769

MEIEILYKKLIKPFLPTPSHLQHYKLSFFDQIALKEHVPIVLFYANNKFINNFTIDERIKQSLSKVLTHVYPAAGRYDKDECSILCLDQ

GISYTKAKVNCKLNNFLEKAHRDLSLAALFWPHENKYINKSNLMVSPIVTAQVTEFECGGLAVSLSSSHPAMDGFSNIKFLLEWAK

VCKMETPVENINFLRFNLGNVFPTRDISRLFKSTYDPVIEKDIVTKRFIICETIMSRLRKKCIDEARGALTFQPTRVEIITALLWRAFIR

TSTIINGYVRPSLMDLPLNLRSKTSLTQVSNSMGNFRVDVPIKFIPGETKMELHKFIILIRNGVNKVVASCTKASPDEIVSTLVNINN

GSVASPEWGGNDEVDKVSCSSLCNFPFHDIDFGLEKPKLLFFGSKDMQMFWLYDTDIHSQVCVQVDLKENYMKLFECNDDIKAL

TFIHNANL

>Tomato_XP_004243480

MEIEILCTKLIKPCLPTPPHLQRYKLSFFDQISEKEHVPIVLFYANNNNFFNTSTINERIEQSLSKILTHVYPAAGRYDKDECSILCLD

QGVSYTKAKVNCKLYNFLEKSRKDLSLAALFCPHVNKYIDKTNLMVSPIVTAQVTEFECGGLAVSLSFSHPAMDGFSDFKFLFELA

RVCKMETPIENIKFLSFNLGNIFPTRDISRLFKSTFDRVIEKDIIVKRFIVREAAMSRLRKKCIDEARGALDFQPSRIEIITAILWRAFIG

ASTIINGYVRPSLMDFPLNLRSKSYLPQVKNSMGNFRIDVPIKFIPRETKMELHHFVILIRNAVDKVVASCTKASPDEIVSTLVDIYN

ESFEAPEWGGNDEVDKVLCSSLCNFPLQDTDFGLGKPTLVFFGVKDMQMFWLHDTDIRSEVGVQIDLKERYMQSFQCDDDIKD

LTFIGNANL

>Tomato_XP_004251812

MKDSMQVTILSKNLIKPSLPTPKHLKYHKLSFFDQVADVAHLPLVLFYPHCKNNSKHEELEESLSRILSHVYPLAGRFAEDDESLIL

CLDQGVTYIKAKVNCTLDDLLQQTKKDLDLALSFWPQGTMDVDDSNLFVTPLMVVQVTTFECGGLALAISIAHPVMDGCTTFKI

LYEWTKVCKFGTPSKEISFMNFNVGALFPYKHDLSTLLEPPVDEGKRKDSKLIARKFVFEKDAISRLREKFDSISESLGFKPSRVEMI

TALLWRSLIRSTKSAKLKRSVMSFPLNLRGKVADFPEITDSFGNLIIEVPIKFEHDDETKIESLHQIVKLIKESVKVINNKCVKATPDEI

ISLVIDLYKDSYSGLEWGGDDEVMNFTSSSLSRFPIQKTDFGWGKPSLMHFGSRHNQVLWLYDTECETGIVVQMDLEKKHMDKL

VCDQDIIDFAKF

>Tomato_XP_004253179

MELNLEFISTKLIKPSIPTPPHLKNYKLSFFDQLAEREHMPLLLFYPYGNNNDIGDDLFDQKLEKSLSRILSHVYPAAGRLSRDRFSI

DCLDQGVTFTKAKVNCQFNDFIDQVQKDLNLALFFFPRDIQDLKDVDFDSTPPMVVQVTKFECGGIAMSISASHLVMDGFSNFK

FVYEWAKVCKFEIPDDEIDFMSFDFGEILPARDLSRIFPNRVHPVESEERFIANRFFITEQTISSLRDKLTGAIDSGELCFKPSRVEIIT

AILWRALIRVSEAKHGYLRRSLVFFPVNLRGRISLPLKENAFGNYVMDAPIMFVPEKNKMELHDFVTLIRNSVQKAIDACAIGTAD

DIIANVADSYKEIFASKEWGTDNDEVDKCIISSLCKFPMKDADFGRGKPSLMHFGLRNFHSCWMYDAECGSICVQVDLKDSYMS

LFECQSDIKAFTNVLGNQERIQLQPLL

>Tomato_XP_010323318

MESKVLTKIQILSKSIIKSNHDNNVDHPKIYKLSFFDQFALQMHVPCVLFYPLKNPTFTKTPIIHEQFQQSLSKLLSHVYPASGRFSS

DGQSINCHDEGVLYIKAKVDSQFCDFLKDAQKDIDLALNFCPKVDRNDSNLSMTPLVVVQVTEFACGKGLALCVSSEHAVIDGFT

ALKFVYEWSKVSKMGINKINCFTFDDFGTIFPPTSDSHLLKRVESPRDDPNHDFPEMVARRFVINQSVISKLREHVGVVHIRPSRVE

LVIAFLWRALINVYRCKSNGRLRPCLLSVPVNLRGKIDFPRYENSFGNFAIEVPVKFIPGETGMELKDILLLIKDVIQKINVSFAKSSD

DIYSLASKFHKEIQEWEENEQVDVCMASSLCRFPINEADFGWGKPCLLSFGLRRSDMFWLYDTPCGSGIIVQVDLKKDYMDMFG

CDRDLLSLTCE

>Tomato_XP_004252028

MEIKILCTKLIKPFLPTPPHLQHYKLSFFDQISEKEHVSMVFFFHNYNNIDMDERLEQSLSKILTHVYPAGGRYNEKDKYCSILCVD

QGVFYTKAKTNGTLDNFLNKARNDLGHAALFSPHVNKNIDETNFMVSPIVTIQVTEFECGGVAISISTSHPAMDGFSDFQFISEW

AKVCRIGTPIDKINILSFNMGDIFPTRDITGIFKSTPTPIIQQDIVVKRIVIHEDVMSRLRKKCTFSTFQPSRVEIITAILWRAFIRATAII

NGYLRPSLLDFPMNMRSKITFLPQVKNSYGNFMIGVPVKFIPGENKMELHDFIMLIRNAVNKIVASCKKANSPDEIVATLVDSYNV

SFRSPEWGGNDEVDKVMCTSICKFPVHDSDFGLGKPNLIFFGMKDTQMFWLYDIGPEIVVQVDLKERCMQLFDREDDIKDLIFIR

DAKL

>Tomato_XP_010323479

MESKSLTKIQILSKNIIKSNHVNDVDHPNNYKLSFFDQFACQMHVPCFLFYPIKYSTSPKISIIHEQLQQSLSKLLSHVYPASGRFSS

DAQSINCHDEGVLYIKAKVDSQFCDFLKDAQKDIDLALNFCPKINRNDSNLSLTPLVVVQVTEFACGKGLALSLSAEHAVIDGFTA

LKFVYEWSKVSKMGINKINCFTFDDFGTIFPPTSDNHLLKRVESPRDDHNHDFPEMVARRFVINQSVISKLREHVGVVHIRPSRVE

LVIAFLWRALINVYRCKSNGRLRPCLLSVPVNLRGKIDFPRYENSFGNFAIEVPVKFIPGETGMELKDILLLIKDVIQKTNVSFVKSSD

NIYSLASKFHEEIKEWEENEQVDVCMASSLCRFPINEADFGWGKPCLLSFGLRRSDMFWLYDTQCGSGIVLQVDLKKEYMDMFG

CDKDVLSFIFDE

>Tomato_NP_001266253

MNCYIEIQSRKMVKPSAPTPDNLRRLKLSLFDQMDIGAYVPIVFNYLPNSTSSYDHDDKLEKSLSETLTKFYPFAGRFRKGIDPFSI

DCNDEGIEYVRTKVNADDLAQYLRGQAHNDIESSLIDLLPVMHRLPSSPLFGVQVNVFNNGGVTIGIQILHMVSDAFTLVKFVNE

WAHTTLTGTMPLDNPGFGQLPWLFPARALPFPLPDFNTTTAPNYKNVTKRFLFDALAIENLRNTIKANDMMMKQPSRVVVVMS

LIWKVLTHISSAKNNGNSRDSSLVFVVNLRGKLSCTAPSLEHVVGNCVIPATANKEGDEARRKDDELNDFVKLVRNTIRDTCEAIG

KAESVDDISSLAFNNLTKCIEKILHGDEMDFYSCSSWCGFPWYEADFGWGKPFWVSSVSFGHHGVTNLMDTKDGDGIQVTICLK

ENDMIEFERDPHILSSTSKLAFHSLG

>Tomato_NP_001266195

MACRLDIEIQSRKLLKPSASTPDNLRRLKLSLFDQLALRTYIPVLFNYLPSSSSTSYDDELEKSLAETLTKFYPFAGRFAKDIDPFSID

CNDEGVEYVQTKVNADDLAQFLRGQAHNDSESSLIDLLPIKDVEPSSPSSPLFGVQVNVFNNGGVTIGIQISHIVADAFTMATFVN

EWAHTCLTGRTVSNNPGFGQLSLLFPAKVLQFPSPSPDLNTNTTTTGPNYKIVTRRFVFDALAIENLRKTIKDNDMMMKQPSRVV

VIMSLMWKVLTHISSAKNNGNSRDSSLGFPINMRGKLSCTAPSLEHALGNYGMMGIADRKARRKDDELNDFVKLVGNTIWNTC

EAIGKAESVDDISSLAFNNHIKGVEKLLQEDKMDVYGTTSWCKLPWYEADFGWGKPFWVSPVGLNLIEGAILMDTKDGNGVQLT

ICLKEKNMTEFEKHLHIFSSTPILG

>Tomato_XP_010314380

MACRLDIEIQSRKLLKPSASXPDNLRRLKLSLFDELALRIYIPILFNYLPSSSSTSYDDDKLEKSLAETLTKFYPFAGRFRKDIDPFSIE

CNDEGVEYVQTKVNADDLAQYLRGQAHYDIESSLIDLLPIKDVEPLSPSSPLFGVQVNVFNNGGVTIGIQISHIVADAFTMATFVN

EWAHTCLTGRTISNNPGFGQLSLLFPAKVLQFPSPSPDLNANTTTTGPNYKIVTRRFVFDALAIENLRKTIKDNDMMMKQPSRVV

VIMSLMWKVLTHISSAXNYGNSRDSSLGFPINMRGKLSSTAPSLEHALGNYVMLGIADRKARRKDIELNDFVKLVGNTIRDTCEAI

GKAESVDDISSLAFNNHIKGVEKLLQGDKMDVYVTTRWCKFPWYEADFGWGKPFWVSPVGVNLIEGAIILMDTKDGNGVQLTIC

LKEKDMTEFEKHLHIFSSTPILG

>Tomato_NP_001266200

MVMAKLDIEIQTRKILKPSAPTPDNLRRLKISLFDQLARSAYVSIVFNYLPSSSSSYDDDKLEKSLAETLTKFYPFAGRLAKDDPFSI

DCNDEGVEYVRTKVNADDLAQFLGKDDDDIESSLIDLLPIKDVELSSPSSPLFGVQVNVFNNGGVSIGIQISHFLADAFTLATFVNE

WAHTNTLSSMPQDNNDLHKFGDLSSLFPPKMLQLPSFDPNTSSTTTTVPSYKNVTKRFVFDASAIESLKKTIKDDSSMMRKPTRL

VVVMSLLWKVLARISSAKNGNSRDSCFGFVISFRGKVSCIPSTEHVLGTFSIPEIANMEGDVARKDELNGFVKLVGNRIGETFAAID

KASKVDDIYSLTLNNQIKVIEKFVQRDKMDFYGTTSWCKLPWYETDFGWGKPFWVTPVSFRIYEQTTLMDTKDGDGIEIIVTMKE

NDMTEFERDPHILSSTSKLTFG

>Petunia_Peaxi162Scf00160g00833

MSQTNQNLNGSCFQIEILNEKLIKPSLPTPNNLNCYKLSFFDQLAPNFAVPLLYFYPTVPPEKSNLQSAESTHAQLQNSLSETLTKF

YPLAGRFSEDGTSIECHDQGVIYLEAKVNGQLNEFLDNAYKNSDLVKIFVPPIRIRTAELPYRPMMAIQATMFECGGLALAVQIVH

TLGDGFSGCAVTDEWAKVSRMEKSNARTLQFRSDLADVFPPKDNIFDMVKKGRPRGYEMKIVTRIFMFDEVAISKLKENVNKSLS

YSSRVEVVTALIWRCLMRVVRFRVGEDQNLFGNFYLDIPIKCVPSHDNQDLELHEIVTLIRDTKNKILSKIANASSEEIISLVIESTNKI

REGYNDDEIDLYPTSSLCRFPLNECDFGWAKPTWVSRINVPFQMFFLMDSTTGIEVRVCLNEDDMLKLETDIDIVEFSSVPK

>Petunia_Peaxi162Scf00046g00121

MGSEFFNLKDSMQVKILSKSLIKPSKPTPEHLQNYKLSFFDQVADLAHMPLVLFYPNCNNSISEEKLEESFSRILTHVYPVAGRFTE

DESSVLCLDQGVPYIKATTNCQLDDFLQQGKKDLDLALYFWPDGIMDVDDTNIFMTPLMIVQVTKFECGGLALSISSAHPAMDG

FTAFTFVHEWSKVCKLGIPSKEINFMSFNLSTLFPARDLSTLLEPPVDEGKRTLSKLIARRFVFDESAISRLRENFSSAIESEDLSFKPS

RVEMITSLLWRSLIRASSAKSGHLKQSIMSFPLNLRGKIAFPEVANSFGNFIIEIPIRFQPNESKMELHDFVKSIRDAVQQTTSKCAK

ATADEIVSVVVNLYKDSYAGLEWGGDSEVENFTCSSLCRFPMQKADFGWGNPSLMHFGSRHSQVFWLHDTECDTGIAVQMDL

QETYMDFFERDQDILSFAKF

>Petunia_Peaxi162Scf00169g00062

MESLQVEIISKKLIKPSSPTPQLLQNYKLSFFDQLAEREHVPLVLFYPQCTSNSIIDEKLEQSLSRMLTHVYPGAGRLSKDGHSIDCL

DQGVTFIKANVNCQFDDFMNQVHKDLNLALVFFPQGIKGLSDNDFDITPFIVAQVTKFQCGGLVLSISASHPVMDGFTNFKFVYE

WAKVCKLGIPSEEINFLSFNFGDIFPARDLSSIFPPRINDPKDLDARFVGKRFVIDQGTISVLRDKLASAIDSGELSFKPSRVEIITAIL

WRALIRVSEAKHGYLRPSLMFFPVNLRGRISLPLKENAFGNFVIDAPILFVPGETKMELHDFVTLIRNSVQKTVDVCAKGSSDDIVL

DVANLYKESFLSPKWGGNNEVDKCMISSLCKFPMQEADFGVGKPKLMHFGLGDYHSCWMYDTECGTGICVQVDLKDTYMHLF

ECDQDIKAFFN

>Petunia_Peaxi162Scf00188g00081

MANLDVKTQIRKPLKPSTPTPNHLQNLKLSLFDQLTPPTYVSLLFHFLPSSEWNSTENTERCDKLQKSLAEALAKFYPLAGRCNKD

DMSIHCNDEGVEYVETKVDADLAQFLHEGPKIELLDDLLPWCCIPPDVNLPSSPLLGIQVNIFNCGGLVIGLQISHILADAFTLSAF

VDEWAHISRSGTTKRCFPSFDHLPSLFPTKVLSESHQFSAPSNIVPPKIVTRRFVFDALSIAKLKNRIDSGDTIVKYSRVEVVMSLIWK

VLVGISSAKHERPKDSGLVFAVNLRGKSNLPSLEHALGNFCTTTIATLEANQSSQELNDLVNLVTSASRDTSVGIGKASIDEIASMFI

KSHTGLVNKLGQQNKMDLYIISSFCRFPWYEADFGWGKPFWVSSLGKPFEIVNLIDTKDGDGIEAWVSLKENDMTEFARDLDILT

CCPPLESSS

>Petunia_Peaxi162Scf00883g00007

MDLKAFLEIEILSKNLIKPSLLTPNHPKSYNLSFFDQFSCQMHVPCFLFYPFTSPSEISTIDQCLEQSLFKILTHIYPAADRFSADGHSI

DCHDQGVTYIKAKANCQLSEFLKEARKDIDLVLNFRPKIEYDVTYNKLATTPLVAVQVTKFDCSGLALCVSTANSIIDGFTALTFIY

EWAKLSKIGTSNKVINCFSFDLGNTFPRSDHLSKILKSPSSSLKPRDIKRVSRRFVVTESAISRLRDKVVDSMNSGVFLSFLLNLPMN

LRGKIDLPRYANSFGNFAIDVPVKIIPGETNMEFQDFIELLKEVKQKTSLSFAKASTSDIFSMATKSHDEIKEWEGNDEVDVFPIYET

DFGWGKPSLASFGLRRSDMFWLYGTKCGTGIIVQVDLKQAYMSRFECDQDVLDFTSK

>Petunia_Peaxi162Scf00299g01145

MAKLEIQLMIRKMLKPSTPTPNHLQSLNLSLFDQLQTRVYVPVLFYYLSSSEWKNTERCDRLQKSLADALTKFYPLAGRFRQDDL

SILCNDEGVEYVETKVNADLAEFLHGEPKLELLDDLLPWRVPPKPDLPSSPLLGVQANIFNCGGLVMAIQISHILIDVFSIATFLKEW

AHVSQTGKTEGCLSSFGHLPALFPTRVLPEHLFSPLSIGGAKIVTKRFVFDALAIAKLKDRIKSSATLTKPTRVVVIMSLLWKVLVGLS

STKHGHSRGSTLSFSVDLRRKSNLPSLQHALGNFCVGAEATLEANQSPKELNDFVNLVGSTSTDISLDIGKASIDDLTSMCVSSNTE

FVNKFGQKDKMDIYLISSWCRFPWYEIDFGWGKPFWVSAVSHPFEVISLIDTKDGDGIEAWVGLKENEMAEFERDPDILTFCPPL

GNLQLQPRIFFGALMQSRMYVPVLYYYLPSCEWKLTEICDKLQKSLADTLTKFYPIAGRFIKDDRSIHCNDKGVEYVETKVNADLV

EFLHEGPEIELLDDLLPWCVPPDTNLPSSPLLGVQVNIFNCGGLVIGIQISHVLADAFTIATFLKEWAHISLTGTIEGCLPSFGHLSSL

YPARVLPENQFSPLSFGGAKIITRRFVFDALAIAKLKDRIDLNATLTKPTRVVVVMSLIWKVLVGISSAKHGPSRDSALIFPVNLRRD

SILPCLQHALGNCCAAAIATLEGNQSKEELTDFVNLVGNASRDTSEGIGKASNNDITSNFDSYSTLGQKDEMDIYVCTSWCKLPW

YEADFGWGKPFWVSDLKKCAELVSLIDTKNGDGIEAWIGLKENDMAQFETDPDILTFCPPPKQQLIPRVG

>Petunia_Peaxi162Scf00538g00001

MAKLQIQIKIRKRLKPSTPTPNHLQRVNLSLFDPPDPSSYIPVLFHYLPSSELNTAENICERCDKLQKSLAEALAKFYPLAGRFNED

DLSIHCNDEGVEYTETKVNADLAEFLNEGPKIELLNDLLPWDIIPTSELPLLGIQVNIFNCGGLVMGIQLSHILADAFTLAAFLNEWS

HIVQTGTTNKGCLSSFGHLSSLFPTRVISRPQYSPPSNIVTKIVTRRFVFDALSIAKLKHKINSSAPFVKSPTRVVAIMSLIWKALVGIS

SAKHVSPRDSSILFAINLRGRSNLPSTERALGNFCFIVNSTVEANQSRKELNDFVNLVGSTIRDTSVGIGQAGIDETSSMLINNNEET

LSKFCMKDEMDVYGMSCWCKFPWYEADFGWGKPFWVSSINYVAENILLLDTKDGDGIEAWVGLKENDMAEFERDPDILTFCLP

GKQQLIPQ

>Petunia_Peaxi162Scf00375g00013

MAKLDIEIKIRKLLKPSTPIPNHLHCLNLSLFDLPNPSLYIPILLHYLPSSELNTAENIRERCDKLQKSLAEALAKFYPLAGRYNKDD

MSIHCNDEGVEYVETKVNADLAEFLNEGPKIELLNDLLPWDIIPKTKTDLPLFAIQVNLFNCGGLVMGIQLSHVLADAFTFATFLN

EWSHIVQTGTANKSCLSSFGHLPSLFPVKTISRHHHSPPSDIVTKIVTKRFVFDALSIEKLKHKINSSAPFVKSSTRVVAVMSLIWKAL

VTISSAKHGRPRDSTILIPVNLRGRLNVPSTKHALGNFCLIPNATAEANQSRKELNDFVNLVGSTIKDISVAIGKAGIDETSSMLVNC

SAEILSKYGRKDEMDIYVITSWCKLPLYEADFGWGKPLWVGGVNGVGENIWLLDTSDGDGIEAWLCLEENDMAEFERDADIRTF

CLPQRHQLIP

>Petunia_Peaxi162Scf00375g00003

MAKLDIEIKIRKLLKPSTPIPNHLHCLNLSLFDLPNPSLYIPILLHYLPSSELNTAENIRERCDKLQKSLAKALAKFYPLAGRYNKDD

MSIYCNDEGVGYVETKVNADLAEFLNEGPKIELLSDLLPWDTIPKSDLPLFAIQVNLFNCGGLVMGILLSHVLGDAFTFATFLNEW

SHIVQTGTTNKGCLSSFGHLPSLFPIKTISRHHYSPPSDIATKFVTRRFVFDALSIERLKQKINSGAPFVKSSTRVVAVMSLIWKALVTI

SSAKHGRPRDSTILIAMNLRGRSNVPSTKHALGNFCLIPNATVEANQSRKELNDFVNLVGSTIRDTSVAIGKAGIDETSSMLVNCS

AEILSKYGRKDEMDIYVISSWCKIPLYEADFGWGKPLWVSGVSGAGETIWLLDTIDGDGIEAWVCLEENDMAELERDHDIRTFCL

P

>Petunia_Peaxi162Scf00529g00012

MTKLEIEIQIRKPLKPSTPTPNHLQTFNLSLLDQMTSRMYVPLLFYYFPSSELIKAENNTERCDKLQKSLAETLAKFYPLAGRCRKD

RLSIHCNDEGVEYVEAKVNADLTNFLHDGPKIELLDDLLPWPVPPESYLPSSPLLGVQVNLFNCGGLVIATQISHILADAFSMATFL

NEWAHNSLTGKTKGCFQSLDHLSSLFPARVLEEHQFPPPPSRGANIVARRFVFDVLSIAKLQDRIHSGATFMKPSRVLLVMSLIWK

VLVGISSARHGRSRDSCSIIAMNMRGKSNIPSSENALGNFCSAAVATLQANQSNDFNDFVNLLRSTSKEKSVGIGKASIDDITSLLL

YRDTRVVHNPCQKDDVDTYVFSSWCRFPWYEVDFGWGKPFWVSTVGFPSGVVILIDTKNGDGIEAWVNLKENDMAEFEGHPEL

LTFCHLHSIP

>Petunia_Peaxi162Scf00305g00081

MLKPSTPTPKHLHRLNLSLFDHPHTNIYVPTLFHYFPSTEWNKTENSSSIHERCDKLQSSLAEALAKFYPLAGRFNEDDISIHCNDE

GVEYIETSVNAVLAEFFNDGPKIELLNDLLPWVVTPKSEFPLLGVQVNIFNCGGLVMGIQFSHILADAFTLATFVNEWSHIVQTGTT

SKGCLSTFGHLPSLFPTRVISRPQYSPPPDIVTKIVTRRFVFDALSIGKLKENIESNGTFMKSSTRVVIVMSLIWKVLVSISSAKHGRPR

DSTILFALNLRGKSNLPSTEHALGNFCLIRNATAEAEKSIKELKDFVNLVGSTIRDTSVGIGNASIDEVSSVLINNNTEILNKFYQKDK

MDIYVITSWCKFPLYEADFGWGKPFWVSGINNTTENIRLFDTKDGGGIEAWVGLKENDMAEFETDPDILTFCPPRKQPLIPQFG

>Petunia_Peaxi162Scf00538g00013

MLKPSTPTPNHLQKLNLSLFDHPNAIYVSLLFHYLPSSEWNEAENTTNERCDKLQSSLAEALAKFYPLAGRFNEDDLSIHCNDEG

AEYIETKVDADLSEFLNEGPKIELLNDLLPWDVAPKSDLPPLRIQVNVFNCGGLVMGIQMSHILADAFTFATFLNEWSHISKTGTT

NKVCLSSFGHLPSIFPTRMISRPQYSPPSDIVTKIVMRRFLFDAFSIEKLKGNIDSSGTFVKSSTRVVVVMSLIWKALVGVSSAKYGRP

RDSTILFAVNLRGKSNLPSTKHALGNFCLVGNATVNANQSRKVLNDFVNLVGSTIRDTSVGIGKASIDETSSMLVNNNAQISNKFC

QKDEIDIYLITSWCKFPWYEADFGWGKPFWVSSINCTTENIILIDAKDGDGIEAWVSLNENDMAEFERDPDILACCQPRKQQLIP

>Petunia_Peaxi162Scf00188g00134

MAKLQIQIKIRKRLKPSTPTPNHLQRLNLSLFDPPNPSLYVPVLLHYLPSSELNTAESIRERCDKLQKSLAEALAKFYPLAGRYNND

DMSIHCNDDGVEYVETQVNADLAEFLNEGPNIKLLNDLLPWDIIPKSDVPLFAIQVNIFNCGGLVMGIQFSHILADAFTLATVLNE

WSHIVQTGTTNKGCLSSFGYLPSLFPMKAISRVVQYSPSPDIVTQFVTRRFVFDALSIEKLKHKINSSAPFVKSSTGVVVVMSLIWKA

LVAISSAKHGHPRDSTILFAMNLRGRSNVPSTEHALGNFCLISNATVEANQSIKELNDFVNLVGGTIRDTSVAIGKAGIDETSSMLV

NSSAELLSKYGRKDEMDIYVITSWCKIPWYEADFGWGKPFWVSCINYDGEVIILLDTKDGDGIEAWVSLKENDMAEFERDPDILT

CCLPQKKQLIPQ

>Petunia_Peaxi162Scf00003g00342

MEVQVLSRKIVKPSKPTPLHLQNLKLSLFDQLSPPVYVSLLFYYSSNGEENAAKSTEKFTLLEQSLSKALTSFYPLAGRFSNDDLLIN

CSDQGVEFVEAQVEKDLAEFLNEGPQIELLNQFMAWDVLPSTLLATSPLLAIQVNMFSCGGLVIGIQCSHQVADAFTLEKFINQW

ACISRTGEKEMSCSSSFGNLASLFPARTLPGPQLPPPRMRNSSKIVTRRFVFDAKAIADLKERILSKDTSIAPSRVVTVIALLWKALV

GASLAKNSYLKNFELRPTMNLRERTVLPIPQQTLGNFWIHGIVHFEANKNGTELHDFAKLMKNAIRETTMNIAKASIDEISAMLIN

DYKEIVDKLANNEKDPAFTTWCRYSWYEADFGWGKPIWVSGIGIPFEVIGLIDTKNCDGIEAWVSLKEEVMVAFEQDPDILSLAVS

PPK

>Petunia_Peaxi162Scf01732g00002

MAKLQIQIKISKTLKPSTPTPNHLQRLNLSLFDYPIPSSYVPMLLHYLPSSELNTAENTSERCDKLQKSLAEALAMFYPLAGRFNEE

DLSIHCNDQGVDYVETKVNADLAEFLNQGPNIELLNDLLPWDIIPKPDLPLLAIQVNIFNCGGLVMGIQFSHILADAFTLATFLNE

WSHIVQTGTTNKGCLSSFGHLPSLFPLRAIPRHKYLPPLDIVTKFVTRRFVFDAPSIEKLKHKINSSSPFVKSSTRVVAVMSLIWKALV

TISSAKHGRPRDSTILFAMNLRGRSNVPSTEHALGNFCLIPNATAKANQSRNELNEFVNLVGRTIRDASVAIGKAGIDETSSMLVN

NSADIFSKYCRKDEMDIYVITSWCKIPWYEADFGWGKPFWVSSVNCAGENIMLLDTIDGDGIEAWIGLEENDMAEFERDPDIMT

FCLPQKHQLIHR

>Petunia_Peaxi162Scf00299g00103

MLKPSTPTPNHLQRLNLSLFDQLAPPIYISMMFYYLPSSERDRAESVEKCAKLQKSLADTLAKFYPLAGRFIKDDLSIHCNDEGVE

YVETKVDADLAEFLHEGLKIELLDDLLPWCVPPKADLPSSPLLGIQVNVFNCGGLVIGIQISHILADSFTLATFLNEWAHTSLTGSTK

DNCIPNFGHLSSLFPTRVLSGPDQGSPPFSTGAKIVTRNFVFDALAITKLKDKLIHSSTAAATLQKLPSRVVVVVSVIWKVLLDISSA

KRGHDSRDCVLFLTVNFRGKSNAPSSEHAVGNFWMTEFAILEAEQSRKELNDFVDAVGTAIRDTSVRIGNTSIEDIPSLFVKNKTK

LGDKRRQRDMIDIHGCTSWCRFPWYEADFGWGKPFWVSSVSQPFEVICLIDTKDGDGIEAWISLKENDMAEFERHPDILTFCSPL

QN

>Petunia_Peaxi162Scf00003g00335

MEVQVLARKIIKPSKPTPLHLQNLKLSLFDQLTPPLYVSILFYYSSNGEENASKSSDEKFTLLEESLSKALTTFYPLAGRFSNDDLSIN

CNDQGVDFVEAHVKKDLAEFLNEGPKLELLHQFFAWDNPPSSLLATSPQLAIQVNMFSCGGLVIGVQSSHLVGDAFTRDKFINH

WACVSRGEKEMSSSSSTFGNLASLFPTRTFPKPQLPPQRTRNTSKIITRRFIFDAKAIANLKERIVSKDTSIERLSRVVAVIALIYKALA

GATLATNGHLKDFLLRPAMNLRGRTVLPVPQHTLGNFVICGMAPFQANKNGIELHDFAKLMSDAIRETTMKMVKASIDEISDLLI

KNYEAIVAKLISNEKDVPAFTSWSRFSWYEADFGWGKPIWVSCKSPSFEIISLFDTKNGDGIEAWVSLREEVMVEFEQDPDILSLAV

CPPK

>Petunia_Peaxi162Scf00299g01143

MNLRERTAAVPISQNAFGNSWIPFIARFTPSGDILEWFDLVLPLSNAVKNTAKVIGKVSSDEISLLAARTFKEVHDEIFGNNGLDVFI

CTSWSKLPVYEADFGWGRPSWVSSSRRLCEMFTLLDSKCGDGIEVEVVSKLGQLDEIDNYFITSWCKLPWYEADSGWEQPFWVS

SVSKSTDIVSLFVTKYGDSLEEWVGLKGNDIAEFKKDLDILSSAWEIRRTLKPSTPTLNHLRNLNLSLFDQLAPCMYVPVLLYYLPSS

ELNYTENTERYEKLQKSLAETSAKFYPLAGRFRKDDFSIHCNDEGVEYVETKVNTDLAECLHEGPKIDLLDDLLPWCVPPKADLES

SPLLGVQVNIFNCGGLVIGIQISHILADAFTLATFVNEWAHISQIGKTKGCLPSFNHLSSLFPAKVVSVPQFSLASSEGSKLVTRRFLF

DALAIEKLKDKIDSSVTFTKPTRVVVVMALLWKVLVGISLSKHGHSRDSSLLFAISLRGKSKLPSSEHALGNFVTTGTSTLEANQSRK

ELKDFVNLVSNTTRDIYVGIGKADIDDVTSMSVNNRTEAVNKLGRKDEMDTCLITSWCKFPWYEADFGWGKPFWVSIVSKSTEVI

TLIDTKTGDGIEAWIGLKENDMAEFERHPDILTLKMLKPSTPTPNHLQSHNLSLFDQLAPRMYVSVLLHYLPSNEWKNTEICDKL

QKSLADTLTKFYPLAGRFKKDDLSIRCNDEGVEYVETKVNANLAEFLREGPKIELLNDLLPWCAPPNEDMTSCPLLGIQASIFNCG

GLVMGIQIAHILADAFTLATFLNEWALISLTGTTNSCLSSFSHLPSLFPKRVLSAPSQSLPLPQECAKIVMKRFVFDALAIAKLKGKV

DSSAAFTKPTRVVVVMSLIWKVLVGISTTKHGRSRHSTLKFVVNMRGKSTLPSTEHALGNFGTTGVATLEANQSRHELNDFVNLV

RSASRDTSETIGKASIEDITSMFVNCDTGVMVNNTCQKDDMDIFISSSWCRFPWYETDFGWGKPFWVSSVSQDAFEVIGLFDTN

DGDGIEAWLDIKIQIRKTLQPSTPTPDNLRNLGLSFFDQMQSRMYVPVLYYYLPSSVWKITEICDKLQTSLADTLTKFYPVAGRFIK

DDLSIHCNDKGVEYVETKVDKYLAEFLHEGPEIDVLDDLLPWCVPPDTNLPSSPLLGVQVNIFNCGGLVIGIQISHV

>Potato_XP_006367986

MFQAKNLTKLNTRLYTMSQITTQNLNGTCIQIEILNEKLIKPSLPTPNHLNSYKLSFFDQIAPNFAVPLLYFYPPVPPENSHLQCVEE

VHKQLQNSLSEVLTKFYPLAGRLSEDGTSIECHDQGVIYLEAKVNCQLNEFLDKAYKDSDLVKIFVPPIRIRLAELPNRPMMAIQAT

MFEHGGLVLAVQVVHTLGDGFSGCAITDEWAKVSRMEKGNVRNLQFRSDLGEVFPPRDNIFEMIKKGRPRGYEMKIATRIFMFD

EIAISKLKENVNKFMSYSSRVEVVTALIWRSLMRVVRLRQGHNRPSMLQFAINLRGRGSPKVVGEDQNFFGNFYLDIPIKYVSSHS

NQDPELHEIVTLIRNAKNKVLSEIANASSEEIFSILIESLNQIREGYNDDEIDLYPTSSLCKFPLNESDFGWAKPIWVSRVNVPFQMFF

LMDSKNGIEARVCLNEDDMIKLEKDVDIVEFSYVPK

>Potato_XP_006351850

MAKLEIQIQMRKMIKPSTPTPNHLRSLKLSFFDQGAPRLYVPILFHYLPTGECNRELGIIEKCEKLQNSLAETLTSFYPLAGRFMED

KFLIHCNDVGVEYVESKVNMNLAEFLRRGPKIELLDDLLPEMDQSSSPLLGVQVNLFNCGGLVMGIQISHIIADAFTLATFLNEWA

NTSLTGTTKDCLPSFGHLSSLFPPRVISAGPQFSLPSIEGPKIITKRFVFDAFAIANLKDRINPSTAIFTRVVVVMSLIWKVLMGISSAK

HGHSRDSSFVFPINLRGKSKLSSLEHAQGNFYVTVAATLEANEMRRELHEFVNAIGSTARNTSLSIGKAASVDEIMSLSINCGTEIIN

KFVQGDAIDIYPSTSWCRFPWYEADFGWGKPMWVSSVGKTFEVISLFDTKDGDGIEAWVSLKENDMLEFERDTQILASTAFDSSI

WIK

>Potato_XP_006350019

MNYSMQVKILSKNLIKPSLPTPQHLKNYKLSFFDQVADVAHLPLVLFYPHCDNNMKNDELEESLSRILTHLYPLAGRFAEDESSII

CLDQGVTYIKATVNCKLDDFLQQANKDVDLALPFWPQGIMDVDETNLFVMPLLVVQVTTFECGGLALAISHAHPAMDGCTTFK

FIYEWAKVCKFGTPSKDINLNLNLGTLFPYKDLTTILEPPVDEGKRKNSKLVARKFVFEEDAILRLREKFDSTTSEGLSFKPSRVEMIT

TLLWRSLIRSTGSTSHLKRSIMAFPINLRGKVAAFPEIANSFGNLIIEIPIKFEHDDETKMESLHHIVKLIRESVQVIISKCVKATPDEIV

SLVIDLYKDSYAGLEWGGDNEVVNFTCSSLCRFPVQKTDFGWGEPSLMHFGSRHSQVFWLYDTECETGIVVQMDLEEKYMDKL

ACDQDIMDFAKF

>Potato_XP_015161035

MEIGFVNENDYIKVEILCTKLIKPSSPTPSHNQCYKLSFFDQIAEREHIPIVLFYPYNYFNSPTIDERLEKSLSKVLTHVYPAAGRYDK

DECSILCLDQGVSYTKAKVNCKLDNFLEKTHKHLSLATLFWPHENKNVDQNNFMVSPIVTIQVTKFECGGLALSFSVSHPAIDGFT

GLNFLFGWGKVCRLGTPIDKINFLSFNLGNIFPTRDISSLFKSTQVANREENIVVKRFVVREAALSRLRKQCIDESGGALNFQPSRVE

IITAILWRALIRISAARNGYFRSSLMDLPLNLRSKSSLPQVNNSMGNFRIDIPIKFIPGETKMKLHNFIILIRNTVNKVVASCTNASPD

EIVSTLVNIYNESFEAPEWGGNDEVDKVACSSICKFPLQDIDFGLGKPSLVYFGLKDMEIFWLYDTDCHTEVGVQLDLKESCMQLF

ECDNDIKALMFIRDAKL

>Potato_XP_006342686

MELNLEFISTKLIKPSIPTPQHLKNYKLSFFDQLAEREHMPLLLFYPHGNNDDKFDEKLEKSLSRILSHVYPAAGRLSRDRCSIDCL

DQGVKFTKAKVNCQFNDFINQVQNDLNLALFFFPRDIQDLKDADFDSTPPMVVQVTKFECGGIAISISASHPVMDGFSNFKFVYE

WAKVCKFGIPADQIDFMSFDFGEILPARDLSRIFPNRVHPVELEAKFIAKRFFINEQTISSLRDKFTGAIDSGELCFKPSRVEIITAILW

RALIRVSEAKHGYLRRSLVFFPVNLRGKISLPLKENAFGNYVMDAPIQFVPEKNKMELHDFVTLIRNSVQKAIDACAIGSADDIIAT

VAKSYKEIFASKEWGTDNDEVDKCIISSLCKFPMKDADFGRGKPSLMHFGLRNFHSCWMYDAECGSICVQVDLKDSYLSLFECES

DIKAFTNGNQERIQLQPLL

>Potato_XP_015159595

MEIEILCTKLIKPFLPTPPHLQHYNLSFFDQISEKEHVPIVLFYANNNFINSSTIDERIEQSISKILTHVYPAAGRYNKDECSILCLDQG

VSYTKAKVNCKLDNFLEKTRKDLSLASLFWPHVNKNIDKTNLMVSPIVTAQVTVFECGGLAVSLSGSHPAMDGFSTFKFLFEWAK

VCRFETPVEKINFLSFNLGNIFPTREISGLFKSTYIPSIHQDIIVKKFVVREAAMSRLRKKCIDEACGALTFQPSRVEIITSLLWRAFIGS

SAIINGYVRPSLMDFPLNLRSKSSLPQVNNSMGNFRIDIPIKFIPGETKMELHHFIILIRDTVNKVVASCTKASPDEIVSTLVNIYNESF

EAPEWGGNDEVDKVLCSSLCKFPLQDIDFGLGKPTLLFLGVKDMQMFWLHDTDIHSEVGVQMDLKESYMQLFKCDDDIKALTFI

RNANL

>Potato_XP_006366967

MESKVLTKINILSKNLIRSFPTSNHDNDVDHPKFYKLSFFDQFALQMHVPCVLFYPIKYSTFTKISIIHERLEQSLSKLLTHVYPASGR

FTSDGQSINCHDEGVLFIKAKVDSQFCDFLKDAKKDIDLALDFCPKIDRSVSNLSITPLVVVQVTEFACGTGIALSVSSEHAVIDGFT

ALKFVYEWSKVSKMGINIHSDKINCFTFDDFGTIFPPTSDDHLLKRVKSPRDDHYHDLAEMVARRFVINQSVISTLRENVGVVYFR

PSRVELVIAFLWRALINVYRRKNNGRLRPCLLSVPVNLRGKVDSPRYENSFGNFALEVPVKFIPGETGMELQDILLLIKDVIQKTNVS

FAKSSDDIYSLASKFHEEIQEWEENEQVDVCMASSLCRFPINEADFGWGKPCLLSFGLRRSDMFWLYDTQCGTEIIVQADLKKDY

MDMFGCDKDVLSFTCDE

>Potato_XP_006351755

MAKLEIQIHSRKMLKPSIPTPNHLQTLKLSFFDQMVPRAYVPVLFHYLPSCSTTTERCDKLQKSLAQILTNFYPIAGRFNEDEFSIQ

CNDEGVEYVQTKVSADLAEFLHQGPNTELLNDFLPWPSAVPPYSDLPSSPLLGVQVNLFNCGGLVMGIQISHIVADAFTLATFVK

EWAHTCLTGTTKDCLPSFGHLSSLFPTRVLSGIQYSPPSNRGTKSFTRRFVFDALAITKLKNTIKYSSAIKPTRVMVVMSLIWKVLAG

ISTAKNGHSRGSSFLFPINLRGKSKLPSLEHALGNGTMFGIADLEASQELNHELVGNTIRDTCVSIGKAARVDDVFCLVVNNQIKA

VDMLCQGDEMDIYSCTSWCGFSWYEADFGWGKPFWVSSVSLDAIEVIMLMDTKDSDGVEAWVSLKENDMAEFERDDDILTFC

SPLPK

>Potato_XP_006367698

MESKVLTKINILSKNLIRPFPTSNHDNDVDHPKNYKLSFFDQFALQMHVPCVLFYPIKYSTFTKISIIHERLEQSLSKLLAHVYPASG

RFASDGQSINCHDEGVLYIKAKVDSQFCDFLKDAQKDIDLALNFCPKIDRSVSNLSITPLVVVQVTEFACGTGLALSLSTEHAVIDG

FTALKFIYEWSKVSKMGINFHSDTINCFTFDDFDTIFPPTTDNHLLKRVKPPRDDHYHDFTQMVARRFVINQSAISKLRENVGVVY

FKPSRVELVIAFLWRTLINISQHKNNGRLRPCLLSVPVNLRGKIEFPRYEKSFGNFAIEVPVKFIPGETRMELQDILLLIKDVIQKTNIS

FAKSNDDIYSLASKFHEEIQEWEENEQVDVCMASSLCRFPINEADFGWGNPCLLSFGLRRSDMFWLYDTPCGTGIIVQADLKEDY

MDMFGCDKDVLSYACDE

>Potato_XP_006351852

MAKLEIEIQTRIMLKPSTPTPNHLRTLKLSLFDELAPHAYVPVLFHYLPSNTEGTITERCDKLQKSLAQTLTKFFPLAGRLIEGEFSIH

CNDKGIEYVETKANANLAEFLHQGPNTELLNDFLPWPSTVPPYSDLPSTPLLAVQVNLFNCGGLVIAIQISHIVADAFTLATFLNE

WANTSLTGTAKDCLPSFGHLSSLFPTTLRSGTQFLPPSNRGPKTITRRFEFDALAITKLKNTIEDSSAIRPTRVVVVMSLIWKVLVGIS

TTKHGHSRDSSFVVLINMRGKSNLPCLEDDALGNCTMSGIANLEAEKELNHELVGNTIRDTCAGIGKVASVDDISSLVVNNHTKV

NEKFLQGEKMDIYVCSSWCGFPWYKVNFGWGKPFWVSSVSFDALELICLMDTKNGDGIEAWVSLKENVMAEFERDADILTFCSP

LSK

>Potato_XP_006351849

MANLEKIRKMLKPSTPTPNHLRTLKLSFFDQLALPVYVPILFYYLPSVDIISCDKLQNFLAETLTKFYPLAGRFSEDDDELSIHCNDE

GVEPPKIDLLPEMDHQPSCPLLGIQVNLFNCGGLVIGTYISHKIADAFTLATFVKEWAYTSFTGTLKAGSLPSFDHLSSLFPTRVLSE

TTQFPSYIINTTRPKIVTRRFIFDALAIANLKNTIEDSTAIRPIRVVLIMSLIWKVLVGISTAKNGHSRDSSLLFLINLRGKSNLPSLDND

ALGNCTMYGIANYMEARKDHELNDFVKLVGNTIGDTCVAIGKAESVDDISSLIVNNQRKVIEKFAQGDEIDVYPSTSWCRFCWYE

TDFGWGKPFWVSLVECDAFEGISLMDTKDGDGIEAWVSLKENDMAQFERYPNILSSTSKLAFPFLSIN

>Potato_XP_006351754

MADIEIKSRKMLKPSASTSDNLRILKLSLFDQLAPRSYVPILFNYLPSSTSSYDKLEKSLAETLTKFYPFAGRFGKDDPFSIDCNDEG

VEYVQTKVNADDLPEFLRRQANDIESSLLHLLPVMVHMPSSPLLGVQVNVFNNGEIAIGIKVSHIIADAFTIATFVNEWAHTCLITG

TISTQDTTNNNFFPSFGQLSSLFPARVLQLPSPPNTSTTDPEIVTRRFVFDALTIENLRKTIKDDSTTDDDMIKQPSRVVVVMSLIWK

VLTHISSAKNGNSRNSSLGFAISFRGKLSCVPSLEHALGNYVTIAIANLEASDHSRKDDQLNDFVKLVGNTIRDTCVAIGKASSVDD

ISSLTVNNWKNGVEKLFQGDTMNTYITTSWCKLPWYEADFGWGKPFWVTRVGFNAIEGVFLMDTKDGNGIQVTVCLKEKKMTE

FEKHLDILSSTPIVG

>Potato_XP_015159603

MDERLEQSLSKVLTHVYPAAGRYDDKDEYCSILCLDQGVSYTKAKTNCTLDNFLDKSRNDFGDAALFSPHVNKNIDETNFMVSP

IVTAQVTKFECGGLAISISTSHPAMDGFSVFQFISEWAKVCRMGTSVDKINFLSFNMSDIFPTRDISQLFKSTPIPIIQQDIVVKRIVIR

EAVMSRLTKKCIDESNGGLTFQPSRVEIITSILWRAFIRATTNINGYFRPSLLDFPMNMRSKITFLPQVKNSFGNFMIGVPVKFIPGE

TKMELHHFIILIRNAVNKIIASCKKTNSPDEIVSTLVNSYNESFRSLEWGGNDEVDKVMCTSICKFPVHDSDFGLGKPNLMCFGMK

DTQMFWLHDIGPEIGVQVDLKESCMQLFDLDDDIKDLIFNCDAKL

>Potato_XP_015162131

MEVEIISTKFIKPSSPTPNHLQNYKLGFFDQTTDETHLPLVFFYPPTNNINFSAHEEQLEQSLSRILTHVYPXXCWPQDSWNVDAS

NLFAMPVVIIQITEFECGGLALSMSHAHIAMDGYSTFTVINEWSKVCRLEIPVEKIDFMSFNLVDVFPTRDLSKLLLPRVPPLDRVES

KLVAKRLYINEDSISRLRKEVGGDLCTFKPSRVEMIMALLWRALIRASEKKHGYLRLYPPTNNINFSAHEEQLEQSLSRILTHVYPIS
GRFTEDNSISCQDQGVKFIKAKVNSKLNEFLEKARKDVNLSLLCWPQDSWNVDASNLFAMPVVIIQITEFECGGLALSMSHAHIA
MDGYSTFTVINEWSKVCRLEIPVEKIDFMSFNLVDVFPSRDLSKLLLPRVPLEDRVESKLVAKRLNINEDSISRLRKEVGGDLCTFKP
SRVEMIMALLWRVLIRASEKKHGYLRRSLMNIPINLRTRLISLPQVEKSFGNLGVDAPLKFIPGENKMELHKFVTLIHDTVKETIITC
DKTSPEDIVSAVSNIYNESFLAQDWGGSDEVDRYTSSSLCKFPIQEADFGWGKPCLMHFGSRHDQVCWLYDAECGNGICVQVDL
KEDYMHLFECDNDIKDFFQF

>Potato_XP_015159601

MDERLEQSLSKVLTHVYPAAGRYDDKDECCSILCLDQGISYTKAMTNGTLDNFLDKARNDFGHAALFSPHVNKNINETNFMVSP
IVTIQVTKFECGGLAISISTSHPAMDGFSDFQFISEWAKVCRMGTSVDKINFLSFNMGDIFPTRDVSELFKSTPIPVIQQDIVVKKIVI
REPVMSRLRKKCIDESDGALIFQPSRVEIITALLWRAFIRATTIINGYLRPSLLDFPMNMRSKITFLPQVKNSFGNFMIGVPVKFIPGE
TKMELHHFIILIRNAVNKIVALCKNANSPDEIVSMLVNSYNESFRSPEWGGNDEVDKVMCTSICKFPVHDSDFGLGKSNLIFFGMK
DTQMFWLHDIGPEIGVQVDLKESCMQLFDLDDDIKDLIFIRDAKL

>Potato_XP_006351696

MLSLFDQLAPRTYIPILFNYLPSSSAISFHSFDKLEKSLAETLTKFYHFAGRFGKDDPLISIDCNDEGVEYVQTKVNADDLPEFLHRQ
ANDIESSLLHLILPVMVHMPSSPLLGVQVNVFNNGGIAIGIKISHNIADAFTIATFVNEWAHTCLTGTISTHNYLPSFGQLSSLFPAR
LMLQLPSPSTTGPENIIVTRRFVFDAXXXXXLRKTIKDDSTTDDDMIKQPSRVVVMMSLIWKVLTHISSAKNGNSRNSSLGFAINF
RGKLSCVPSLEHALGNYVTIAIANMEADEARKNQLNDFVKFVGNTIRDTCVAIGKAASSIDDISSLTVNNWKLCVENLLQEDKMD
GYITSSWCKLPWYEADFGWGKPFWVSRVGFNDIEGAFLMDTKDGNGIQLIVCLKEKNMTEFEKHLDNLSSTPIVG

>Potato_XP_015159606

MDIGFGNQKDCIKVEILWTKLIKPCLPTPPHLQCYKLSFFDQLSEKEHVPFLLFYANNNFINNSTIDERLEQSLSKILTHVYPAAGRY
DKDECSILCLDQGVSYTKAKVNCKLDNFLEKTRKDLGLAGLFWPHENKNVDETNLMVSPIVIAQVTEFECGGLAISLSGSHPAMD
GFSDFKFLFEWAKVCRMGTPVEEINFLSFNLSNIFPTRDISGLFKSTYDPVIDKNIIVKRFVIREAAMSRLRKKYMDESDGGLTFQPS
RVEIITALLWRALIRISTTIHGYVRPSLMDFPLNLRSKNLYHKCTILWGILESMFR

>Pepper_XP_016552461

MSQITKQNMNGTCIEIEILNEKLIKPSLPTPNHLNSYKLSFFDQIAPNFAVPLLYFYPPIPPENSQLQSAESVHKRLQNSLSEVLTKFY
PLAGRLSEDGTSIECHDQGVVYLEAKVNCQLNEFLDKAYKDTDLVKLFVPPIRIRLAELPSRPMMAIQVTMFEHGGLALAVQMV
HTLGDGFSGCAVTDEWAKVSRMEKGNARNLQFRSDLEKVFPPKDNIFEMIKKGRPRGYEMKIATRIFMFDEGAISKLKENVNKSL
SYSSRVEVVTALIWRSLMRVVRLRQGHNRPSMLQFAINLRGRGSTTLVGEDQNFFGNFYLDIPIKYMPSHSNQDPELHEIVTLIRE

AKNKILSNIANASSEEIFSILIESLNQIREGYNDDEIGLYPTSSLCKFPLNECDFGWAKPIWVSRVNVPFQMFFLMDSKNGIEARVCL NEDDMINLVKDVDIVEFSYVPE

>Pepper_XP_016552370

MAFEKENMQVEIVSTKFIKPSSPTPNHLQNYKLSFFDQIADEAHLPLVLFYPPTNNINYASHEQQVEQSLSRILTHVYPMAGRINE DIDSICCQDQGVKFVKAKVNGKLNEFLEKAHKDVNLALLCWPQDTWDVDDTNLSIMPIVIVQITEFECGGLALSVSHAHTAMDG FTIFYIIHEWSKVCRLEIPVEKIDFLRFDLTDTFPSRDLSKLLLPRAPDEDRVEANLVAKRLYINEDSISRLREEVGDLCFKPSRVEMIT ALLWRALIRASEKKHGHLRRSLIGVPINLRPRLSSLPQVEKTFGNLVIEAPAKFVPGENNMELREFVKLIRDAVTETITACDKSTPDE VVAAVAAIYNGSFVAPEWGGSPEEVDMFASSSLCRFPIQEADFGWGNPCLMHFGSRHNQVCWLYDAECGNGICVQVELKEAIV KLFECEDDIKNFFEF

>Pepper_XP_016549890

MELNLEIISTKFIKPSSPTPEHLKNYKLSFFDQLAEREHMPLLLFYPRGDESLIADLSFDERLEKSLSRVLTHVYPAAGRLSNDGLSID CLDQGVKFIKAKVNCEFNDFINQVQNDLNLAPLFFPEDIRDLKDADFNTTPPMVVQVTEFECGGIAVSISASHPVMDGFTNFKFV YEWSKVCKFGIPSEEINFLSFDFGEIFPARDLSSIFPPRVHPQDSEEKIIGKRFCIDEVTISGLRDKLAKAIDSGELFFKPSRVEIITAILW RALIRVSEAKHGYLRPSLVFFPVNIRGKILLPLKGNAFGNYVMDAPILFVPGKNKMELHDFVTLIRSSVQKAIDACVIGSSDDIVAN VANSYKEIFMSKDWGIGNDEVDRSVISSLCKFPMKDADFGRGKPSLMHFGLRDFHSCWMYDGENGSICVQVDLKDPYMRLFER DSDIKTFTKFY

>Pepper_XP_016579327

MESKALRNKIKILSKNLIKPSISSPTSKNEHHPKNYKLSFFDQFACQIHVPCVLLYPLKYSMFSFTKNSIIHEQLEQSLSKLLTHVYTA AGRFAVDGQSIDCHDEGVLYIKAKVECKLDNFLKDAQKDIDLAMRFCPKIDRDASNLSTTPLVVVQVTEFDCGGLALSVSMKHA VIDGFTALKFVYEWSKVSKTGIISDKDCFTYDDLGTIFPARDNHLSKIVKSPRDNDHEFVETISRKFVIYESAISRLREIVGVACFKPS RVELVIALFWRSLINVSQRKNNGCLRPSLLIVPTNLRDKIHFPKYKNSFGNFAIEVPVKFIPGETRMELQDIVQLLRDVIQKTSISFAK ASDDIIFSSAAKFHEEIQEWKKNEQVDVCMASSLCRFPINELDFGWGKPCLTTFGLRRRDMFWLYDTECGTGIVVQADLKQGYM NMFQCDQGVLTFTCQ

>Pepper_XP_016541551

MNIHVKILSKSLVKPSTPTPDHLRIYKLSFFDQVADLAHLPLVLFYPHCNNNPKHEELEESLSRILSHVYPLAGRFTEDESSILCLDQ GVTYIKATVNCKLDDFLQQANNDLDLALPFWPHGIMDVDETNLFVTPPMVVQVTTFECGGLALAISSAHPAMDGFAAFTLVYE WAKVCKFFIPSKEINFMSFNLGTLVPTKDLTAILEPPVCEGKRTQSKLIAKKFIFDEVSISKLREEFDSSNVLSFKPSRVEMITSLLWRS LIRAARSPYLKRSVMSFPLNLRGKVVAYPEIASSFGNLIIEIPIKFEHDDETKVESLHHIVKLIRESVQETTSYCAKATPDEIVSLVVNL

YNDGYAGFEWGGDKEVVNFTCSSLCRFPIQKTDFGWGKPSLMHFGSRHSQVFWLYDTPCETSIVVQMDLEEMYLDNLVRDQDI

MAFAKF

>Pepper_XP_016581959

MAKLDIQIQKKKMIKPSTPTLNHLRTLKLSYFDQGAPRMYVPILFHYLPSSVGITERCDKLQKSLAEMLTMFYPLAGRFSEDEFSIL

CNDEGVEYVETKVNTDLAEFLHQGPKNIELLDDLLPTDLPSSPLLGIQVNLFNCGGLVMGIQISHILADGFTLGTFVKEWARISLTG

MTKGCLPISFGGLSSLFPTRVQSGPQFAAPSNRDPNIVTKRFVFDALAIAKLRNRTNSSATFRRPTRVVAVMSLIWKVLVGISLAKH

GHARDSHLLFPINLRGKSNIPSLEHALGNFYVTVVAALEANKSRKELTDFVSVVGGKTRDIAAGFSNANIDDITSACVTYGTEVVS

KLGQKDVDNYPSTSWCGFPWYGADFGWGKPCWVSSVSKNYEGISLLDTKNGDGIEAWVSLKEDEMAEFERDPEILSSTSNRK

>Pepper_XP_016582314

MAKLEIQIHTREVLKPSARTPNHLQNLKLSLFDQLAPRMYISKLFHYLPSSEGIVTETCDKLQKSLAETLTKFYPLAGRFSGDEFSIH

CNDDGVEYVETKVNADLAEFLHLGPKIELLNDLLPWCVSSTTGLPSKPLLGVQVNVFNCGGVVIAIQMSHILADAFTLATFINEWA

HICQGTGTTKDCLPSFDQLPLLFPARVLSGTQSPPPPNSGLKIVTRRFLFDALALAKLKNIIDSSATRPTRVVVIMSLIWKVFAGISSA

KNGYSRNSCLLFPINLRGKSYLPSLENALGNFAMIGIANLEASQSRKELTDFVNLVGNTIRETSAGIVKANSDDDISSIVVNNRTNV

ADKCRQRDEVDIYVCSSWCRLPWYEADFGWGKPFWTSTVSFPAAEIICLMDTKTGDGIEAWVSLTENDMAEFERNHDILTFCPP

LPW

>Pepper_XP_016539279

MDKLEIHIQTRKMLKPSTPTVNHLQSLKLSLFDQLAPRHYEPVLFHYLPSTSDCEEGIIMTERCDKLQKSLAQTLTKYYPLAGRFRE

DELSIHCNDEGVEYVETKVNADLAKFIHQGVPKNIELLNDLLPEMEHVPSSPLLGVQVNVFNCGGVVIAIQMSHILADGFTFATFV

KEWAHISQTGTTEDCLPRFGQLPLLFPTRVLSRTRFSRPPNHGPKIVTRRFVFDALAITKLKNTIDSSATFRKPTRVVVVMSLIWKVL

ADISSAKNGHSRDSSLIFYISLRGKFHSPSLKNALGNCTTFGIADLEASQSRNELTDFVNLVGNTIQDTSVGIGKASVDDVSSLVLN

NQSKVAYKRRQGDTMDIYRCTSWCRFPWYEADFGWGKPFWVSAVSFDIIEIVCLMDTKNGDGIEAWVSLKENVMAEFEKHPDI

LTVCPPLPS

>Pepper_XP_016567819

MSNLEIQIQITEMLKPSTPTPNHLRTLKLSMFDQPDASNYVPMLFHYLPSSEQNNTAEKSDKLKKSLADTLVNFYPLAGRFSRND

LSIHCNDEGAEYVETRVNADLAEFLHQLGPKVELLDHLLPWCNIVPMESTLLLPLLAIQVNIFNCGGLVIGIQISHSISDSYTIATFIK

EWARVSQTGTATKDCLPSFGHLPSLFPPRVVPLEHHQFSSTPAPDIVSPKIVTRRFVFDASVIANLKDRINSSAPFVKPTRVMAVLS

LIWKVLLGISSAKRGHSRDSWLIIPINVRAKSKLPSLEHALGNCTLIGIPTLEANHRQELQDFVNSVGIALRKTLISIGKASIDDIASMV

IDQSREYVNAFGQKDEDIYLSSSWCRFPWYEADFGWGKPFWVSSACRSLEVIILMDTKDGDGIEAWISLKEDDIAELERNIPDILSS

YALKNSI

>Pepper_XP_016549062

MGSLCVDVNDQIQVEIMSKKLIKPSSPTPNNLQNYKLSFFDQLAEHTHTPFVLFYPKKNTSTTTDFMVQQLEESLARTLTHVYPA

AARFHDNKCSIICQDQGVPLIKAKVNRRMDEEFLKQAHNNLDLVLHFWPLINNDVIANNLFAMPIMFVQITIFQCGGIALSVSTA

HPAIDGWTNFTFIYEWSKVCKLGIPSEKINFMSFDLVNIFGPRDITFSDQAKPLDTKLMAKKFVMDEKVLSKLRDKLTSSNNNSEA

LCFKPSRVEMVTAILWRAQLRASRAITGEMKPSVMSFPLNLRGKLKYREAINPFGNFIIDIPITYEPKGTNVMELQHFITLIREEVEKI

VDYCGEASIDEVVEMVGNLYNKCYEGREWGANDDVEEFTCSSLTRFHMQEADFGWGNPNLMHFGSRNNQVFWLYSTQCGNSI

GVQMDLKEKYMDFIQHDQEFLAFTKSV

>Pepper_XP_016578439

MENIKVEILSTRLLKPSLPTPPHLQRYNVSFFDQIANEELVPLVLVYPHCSNNNNSAITDEEMEERLERSFSDILTRVYPAAGRYAD

NDKCCVLCLDQGVPYTKAKVNRKLDDFVKQVACDGHDVTTLLWPHDIKDVDETNLFASPIFTVQITKFECGSLAIAISISHPVMD

GFTTFSTMVEWANACRLRTPIDKINNFLSFNGGDIFPTRDLSRYFKPPIPQEGNKEIKFVSKRFVINKGALLKLREKFSSFIESGALNF

QPSKVEMISATLWRALICASESVNGKLRPSMMGFPLNLRSKTNLPEIKKSVGNLVIDIPVKFVPGQTQLELQHLVTLIRDAVTKLVS

SCAETSPDEIVSHVANLYNASFQAPEWGGNDDVDKFTCSSLCRFPIQDTDFGLGKPSLLFFGLKDMNMFWLHDTVCRTGVGLQI

DLDENHMQLVESNLDVKALIE

>Pepper_XP_016549057

MASEIQVEIISKKIIKPSSPTPNHLQNYKLSFFDQIAEKSHVPFVLFYPYDPIKNKNCTTDSMIQKLEESLSRVLTHVYPAAGRFHEN

KCSIICQDQGVQLIKVKVNRRMDEEFLKQAHNNLDLALQFWPQGSKDVDATKLVATPIMFFQIAIFQCGGITLSTSATHFAIDGW

TNFTFIYEWSKVCKFGTPAEKINFMSFDLANIFGPRDITFSDETEPLVTKLVAKKFVMDEVSVSNLRDELTKCNNSSRSLYFKPSRV

EIITAILWRALLRASHAITGKMKPSVLSFPLSLRGKLKYRETINPFGNFIIEIPINYDTKGGDIELQDFIILIRETVQKTVDFVSEASVDE

VVAMVENLYNKSYGGTDWGASDDVEEFACSSLNRFHMQEADFGWGNPNLMHFGSRDNQVFWLYSTQCGNNIVVQMDLKEK

YMDFIQHDQEFLAFTKLREG

>Pepper_XP_016549058

MASEIQVEIISKKIIKPSSPTPNHLQNYKLSFFDQIAEKSHVPFVLFYPYDPIKNKNCTTDSMIQKLEESLSRVLTHVYPAAGRFHEN

KCSIICQDQGVQLIKVKVNRRMDEEFLKQAHNNLDLALQFWPQGSKDVDATKLVATPIMFFQIAIFQCGGITLSTSATHFAIDGW

TNFTFIYEWSKVCKFGTPAEKINFMSFDLANIFGPRDITFSDETEPLVTKLVAKKFVMDEVSVSNLRDELTKCNNSSRSLYFKPSRV

EIITAILWRALLRASHAITGKMKPSVLSFPLSLRGKLKYRETINPFGNFIIEIPINYEPKGCDIELQDFIILIRETVQKTVDFVSEASVDEV

VAMVENLYNKSYGGTDWGASDDVEEFACSSLNRFHMQEADFGWGNPNLMHFGSRDNQVFWLYSTQCGNNIVVQMDLKEKY

MDFIQHDQEFLAFTKLREG

>Pepper_XP_016567820

MAKLEIQIQTSEMLKPSTPTLNHLRSLNLSMFDQPDASNYAPILFHYLPSNEQNNTAERCDKLKKSLADTLVNFYPLAGRFSRND

LTIHCNDEGAEYVETRVNANLAQFLHQLGPKIELLDHLIPWCNSFPPESTHLPPLLAVQVNIFNCGGLVIGIQISHIITDGFTLGTFM

TEWARVSRTGTATKDCSYGHLSSLFPTRVVPLAHHEFSSLIVGPKIVTRRFVFDAQAIANLKHRINSSAPFTKPNRVMIVLSLIWNV

LVGISSAKRGHSRDSCLIIPINVRAKSKLPSLQHALGNCTLISIPTLEANNRQELHDFANLVGSTLRETLISIGKANIDDIASMVADQS

RELVNAFGQKDEKDIYLSSSWCKFPWYEVDFGWGKPFWVSSACRLLEVIILVDTKEGDGIEAWVSLKEDDIIEFERNIQMVLPRIN

HISSQKITPISRL

>Vitis_XP_010648156

MEVKILSKKLIKPSSPTPSHLRHLTLTPVDRLAPPIKASNIFYYPAKGSNPAVDVERRNRLETSLSEILTRFYPLAGRYVRESHSVDCN

DEGVEYLEAEVEGKLSRLLSRRNEVIEQVIQLAGGEFTNSLASVQVTVFACGGVTIGVRIRHSVVDGFTAAHFSSAWATASRESMD

KVIWPSFDLASFLPVKDLPMVKPRPPPKIFGADKVMTRRFIFDGANISSLKAAACDPSFKREPSRVEAVTALIWRALMVVSRAKHG

RLRTSLASHAMNLREKIVPPLPGICCGNLYTEVPATFMADSGKTELPNLKDLVGLLREVKFKESVSREDVLLTVIKSTNELHEALGK

EDIDVYNFTSWCRFPFYGNDFGWGNPAWMTRCHTPVEMISLQDTECGDGIEAWVTLEKKDMLQFQQDGDIVSFSSNGAP

File S2: SmelAAT and 305E40_aat cDNA and putative peptides sequences.

- cDNA

>*SmAAT*_cDNA

CACTATGAGCCAAATTACAAAACAAAACTTAAATGGTACTTTTATTGAACTTGAAATCTTGAATGAAAAACT
TATAAAACCATCATTACCAACTCCAAATCACCTCAATTCCTACAAATTATCATTTTTTGATCAAATTGCTCCT
AATTTTGCTGTGCCTCTTCTTTACTTCTACCCTCCAGTTCCACCAGAAAAATTGAACCTACAACATGCCGAAG
AAGTTCATAAACAACTACAGAACTCACTGTCTGATGTTCTAACTAAGTTTTATCCACTTGCTGGAAGGTTGTC
TGAAGACGGTACTTCCATTGAATGTCATGACCAAGGGGTTATTTACTTAGAAGCAAAGGTGAATTGCCAATT
GAATGAGTTTCTAGACAAAGCTTACAAAGATAGTGACCTCGTTAAAATCTTTGTACCACCTATAAGAATCAG
GCTAGCTGAATTGCCAAACAGACCAATTATGGCAATTCAGGCCACCATGTTCGAACATGGTGGCCTCGCGCT
AGCCGTGCAAATGGTCCACACATTAGGTGATGGATTCTCAGGTTGCGCAATTACTGATGAATGGGCTAAAGT
TAGTCGTATGGGGAAGGGGAATACAAGAAATTTACAGTTCCGTTCTGATTTAGCAAAGATCTTTCCACCTAA
AGATAATATTTTCGAGATGATTAAAAAAGGTAGGCCTAGAGGATATGAGATGAAAATTGCTACTAGAATTTT
CATTTTTGATGAAATTGCTATATCTAAGTTGAAGGAAAATGTGAACAAGTCTTTGAGTTATTATTCATCAAGA
GTTGAAGTTTTGACTGCACTTATTTGGAGAAGCCTAATGAGGTTGAGGCAGGGTCACAATAGGCCATCCATG
CTACAATTTGCCATAAATTTAAGAGGAAGAGGATCTCCAAAACTACTAGGCGAAGATCAAAACTTCTTTGG
GAACTTTTACCTTGACATTCCAATCAAATGTGTACCATCTCAAAGCAACCAAGATCCAGAATTACATGAAAT
TGTAACCTTAATTAGGAATGCAAAGAACAAAATTCTATCAGACATTGCAAATGCCTCAAGTGAAGAGATTTT
CTCAATATTGATTGAGTCATTGAATCAAATAAGAGAAGGGTATAATGATGATGAAATTGACCTTTATCCAAC
TTCAAGTTTGTGTAGATTTCCTTTAAATGAGTCTGATTTTGGATGGGCTAAACCAATTTGGGTTAGTAGAGTA
AATGTGCCATTTCAAATGTTCTTCTTGATGGATGCAAAAGATGGCATTGAAGCTAGAGTTTGCTTGAATGGA
GATGATATGATTAAATTGGAAAAGGATGTTGACATTGTGGAGTTTAGTTATGTGCCTAAGTAGTGATGGCTG
ATGGGATTGGAAAGGGTGGGCGCGCCGACCCAGCTTTCTTGTACAAAGTTGGCATTATAAGAAAGCATTGCT
TATCAATTTGTTGCAACGAACAGGTCACTATCAGTCAAAATAAAATCATTA

>*305E40aat*_cDNA

CACTATGAGCCAAATTACAAAACAAAACTTAAATGGTACTTTTATTGAACTTAAATCTTGAATGAAAAACTT
ATAAAACCATCATTACCAACTCCAAATCACCTCAATTCCTACAAATTATCATTTTTTGATCAAATTGCTCCTA
ATTTTGCTGTGCCTCTTCTTTACTTCTACCCTCCAGTTCCACCAGAAAAATTGAACCTACAACATGCCGAAGA
AGTTCATAAACAACTACAGAACTCACTGTCTGATGTTCTAACTAAGTTTTATCCACTTGCTGGAAGGTTGTCT
GAAGACGGTACTTCCATTGAATGTCATGACCAAGGGGTTATTTACTTAGAAGCAAAGGTGAATTGCCAATTG

AATGAGTTTCTAGACAAAGCTTACAAAGATAGTGACCTCGTTAAAATCTTTGTACCACCTATAAGAATCAGG
CTAGCTGAATTGCCAAACAGACCAATTATGGCAATTCAGGCCACCATGTTCGAACATGGTGGCCTCGCGCTA
GCCGTGCAAATGGTCCACACATTAGGTGATGGATTCTCAGGTTGCGCAATTACTGATGAATGGGCTAAAGTT
AGTCGTATGGGGAAGGGGAATACAAGAAATTTACAGTTCCGTTCTGATTTAGCAAAGATCTTTCCACCTAAA
GATAATATTTTCGAGATGATTAAAAAAGGTAGGCCTAGAGGATATGAGATGAAAATTGCTACTAGAATTTTC
ATTTTTGATGAAATTGCTATATCTAAGTTGAAGGAAAATGTGAACAAGTCTTTGAGTTATTATTCATCAAGAG
TTGAAGTTTTGACTGCACTTATTTGGAGAAGCCTAATGAGGTTGAGGCAGGGTCACAATAGGCCATCCATGC
TACAATTTGCCATAAATTTAAGAGGAAGAGGATCTCCAAAACTACTAGGCGAAGATCAAAACTTCTTTGGG
AACTTTTACCTTGACATTCCAATCAAATGTGTACCATCTCAAAGCAACCAAGATCCAGAATTACATGAAATT
GTAACCTTAATTAGGAATGCAAAGAACAAAATTCTATCAGACATTGCAAATGCCTCAAGTGAAGAGATTTTC
TCAATATTGATTGAGTCATTGAATCAAATAAGAGAAGGGTATAATGATGATGAAATTGACCTTTATCCAACT
TCAAGTTTGTGTAGATTTCCTTTAAATGAGTCTGATTTTGGATGGGCTAAACCAATTTGGGTTAGTAGAGTAA
ATGTGCCATTTCAAATGTTCTTCTTGATGGATGCAAAAGATGGCATTGAAGCTAGAGTTTGCTTGAATGGAG
ATGATATGATTAAATTGGAAAAGGATGTTGACATTGTGGAGTTTAGTTATGTGCCTAAGTAGTGATGGCTGAT
GGGATTGGAAAGGGTGGGCGCGCCGACCCAGCTTTCTTGTACAAAGTTGGCATTATAAGAAAGCATTGCTTA
TCAATTTGTTGCAACGAACAGGTCACTATCAGTCA

- Peptides

>*SmelAAT*_peptide

MSQITKQNLNGTFIELEILNEKLIKPSLPTPNHLNSYKLSFFDQIAPNFAVPLLYFYPPVPPEKLNLQHAEEVHKQLQ
NSLSDVLTKFYPLAGRLSEDGTSIECHDQGVIYLEAKVNCQLNEFLDKAYKDSDLVKIFVPPIRIRLAELPNRPIMAIQ
ATMFEHGGLALAVQMVHTLGDGFSGCAITDEWAKVSRMGKGNTRNLQFRSDLAKIFPPKDNIFEMIKKGRPRGYE
MKIATRIFIFDEIAISKLKENVNKSLSYYSSRVEVLTALIWRSLMRLRQGHNRPSMLQFAINLRGRGSPKLLGEDQNFF
GNFYLDIPIKCVPSQSNQDPELHEIVTLIRNAKNKILSDIANASSEEIFSILIESLNQIREGYNDDEIDLYPTSSLCRFPLN
ESDFGWAKPIWVSRVNVPFQMFFLMDAKDGIEARVCLNGDDMIKLEKDVDIVEFSYVPK

>*305E40aat*_peptide

MSQITKQNLNGTFIELKS
